# Longitudinal study of the short- and long-term effects of hospitalisation and oral trimethoprim-sulfadiazine administration on the equine faecal microbiome and resistome

**DOI:** 10.1186/s40168-023-01465-6

**Published:** 2023-02-27

**Authors:** Mathijs J. P. Theelen, Roosmarijn E. C. Luiken, Jaap A. Wagenaar, Marianne M. Sloet van Oldruitenborgh-Oosterbaan, John W. A. Rossen, Femke J. W. C. Schaafstra, David A. van Doorn, Aldert L. Zomer

**Affiliations:** 1 Department of Clinical Sciences (Equine Sciences), Faculty of Veterinary Medicine, Utrecht University, Yalelaan 112, 3584 CM Utrecht, the Netherlands; 2 Department of Biomolecular Health Sciences (Infectious Diseases and Immunology), Faculty of Veterinary Medicine, Utrecht University, Yalelaan 1, 3584 CL Utrecht, the Netherlands; 3 WHO Collaborating Centre for Reference and Research on Campylobacter and Antimicrobial Resistance from a One Health Perspective/OIE Reference Laboratory for Campylobacteriosis, Yalelaan 1, 3584 CL Utrecht, the Netherlands; 4 Department of Medical Microbiology and Infection Prevention, University Medical Center Groningen, University of Groningen, Hanzeplein 1, 9713 GZ Groningen, the Netherlands; 5 Department of Pathology, University of Utah School of Medicine, 15 North Medical Drive East, Ste #1100, Salt Lake City, Utah 84112 USA; 6 HAS University of Applied Sciences, Onderwijsboulevard 221, 5223 DE ‘s-Hertogenbosch, the Netherlands; 7 Department of Population Health Sciences (Farm Animal Health), Faculty of Veterinary Medicine, Utrecht University, Yalelaan 7, 3584 CL Utrecht, the Netherlands

**Keywords:** Microbiota, Horse, Antimicrobial resistance, Shotgun metagenomic sequencing, Antimicrobial resistance genes, *sul2*, *tetQ*, *ant6-1a*, *Aph(3”)-lb*, *lnuC*

## Abstract

**Background:**

Hospitalisation and antimicrobial treatment are common in horses and significantly impact the intestinal microbiota. Antimicrobial treatment might also increase levels of resistant bacteria in faeces, which could spread to other ecological compartments, such as the environment, other animals and humans. In this study, we aimed to characterise the short- and long-term effects of transportation, hospitalisation and trimethoprim-sulfadiazine (TMS) administration on the faecal microbiota and resistome of healthy equids.

**Methods:**

In a longitudinal experimental study design, in which the ponies served as their own control, faecal samples were collected from six healthy Welsh ponies at the farm (D0–D13-1), immediately following transportation to the hospital (D13-2), during 7 days of hospitalisation without treatment (D14–D21), during 5 days of oral TMS treatment (D22–D26) and after discharge from the hospital up to 6 months later (D27–D211). After DNA extraction, 16S rRNA gene sequencing was performed on all samples. For resistome analysis, shotgun metagenomic sequencing was performed on selected samples.

**Results:**

Hospitalisation without antimicrobial treatment did not significantly affect microbiota composition. Oral TMS treatment reduced alpha-diversity significantly. Kiritimatiellaeota, Fibrobacteres and Verrucomicrobia significantly decreased in relative abundance, whereas Firmicutes increased. The faecal microbiota composition gradually recovered after discontinuation of TMS treatment and discharge from the hospital and, after 2 weeks, was more similar to pre-treatment composition than to composition during TMS treatment. Six months later, however, microbiota composition still differed significantly from that at the start of the study and Spirochaetes and Verrucomicrobia were less abundant. TMS administration led to a significant (up to 32-fold) and rapid increase in the relative abundance of resistance genes *sul2*, *tetQ*, *ant6-1a*, and *aph(3”)-lb*. *lnuC* significantly decreased directly after treatment. Resistance genes s*ul2* (15-fold) and *tetQ* (six-fold) remained significantly increased 6 months later.

**Conclusions:**

Oral treatment with TMS has a rapid and long-lasting effect on faecal microbiota composition and resistome, making the equine hindgut a reservoir and potential source of resistant bacteria posing a risk to animal and human health through transmission. These findings support the judicious use of antimicrobials to minimise long-term faecal presence, excretion and the spread of antimicrobial resistance in the environment.

Video Abstract

**Supplementary Information:**

The online version contains supplementary material available at 10.1186/s40168-023-01465-6.

## Background

The intestinal microbiota and a well-functioning gastrointestinal tract are essential to equine health [1]. Several host and environmental factors have been identified to affect the equine faecal microbiota composition [2]. Disturbances of the intestinal microbiota are associated with significant health problems in horses, such as colitis [3]. The administration of antimicrobial drugs profoundly affects the intestinal microbiota composition in horses, especially drugs administered orally, and can lead to dysbiosis and clinical disease [4–6]. The short-term effect of antimicrobials on the composition of the faecal microbiota has been studied and microbiota composition seems to recover to a large extent within 25 days post-treatment, although subtle differences could still be observed at this time [5]. How long these changes in microbiota composition persist and whether these have clinical implications is currently unknown.

Antimicrobial resistance is a growing problem in human and veterinary medicine [7–9]. Exposure to antimicrobials might affect the relative abundance of specific genes within the faecal microbiome, especially antimicrobial resistance genes (ARGs). All the ARGs in a certain environment, of both pathogenic and non-pathogenic bacteria, are called the resistome [10]. The use of antimicrobials provides a selection pressure for resistance genes to emerge and potentially persist in bacterial populations and change the resistome [7, 8]. ARGs are often located on mobile genetic elements such as plasmids, facilitating the spread of resistance among bacteria by horizontal gene transfer [11]. Furthermore, bacteria and resistance genes are not restricted to specific ecological compartments (e.g., animals, humans, soil) and can spread easily from one compartment to another [7, 9, 11]. Therefore, a One Health approach is needed to address this problem [8, 9]. In the equine hindgut, the resistome can potentially harm the host’s health in case of infection but can also lead to the spread of resistance genes in the environment by faecal excretion [12]. This is especially relevant as horses’ manure is also used in agriculture [13]. Moreover, horses are kept in close contact with humans, and many of the antimicrobials used to treat infections in horses are also used in human medicine [14]. Therefore, the equine hindgut is a potentially significant reservoir of ARGs, which could be a source of antimicrobial-resistant infections in animals and humans.

Every year, large numbers of horses are hospitalised and even more are treated with antimicrobials in equine hospitals as well as in the field. Trimethoprim-sulfadiazine (TMS) is one of the most widely used antimicrobials in horses. Transportation alone and treatment with antimicrobial drugs have shown to affect faecal microbiota composition [5, 15], but the cumulative effect of transportation to the hospital, hospitalisation, and antimicrobial treatment is currently unknown, and existing work has only focused on short-term effects [5]. Up to date, studies in horses reporting information on the faecal resistome are scarce [12, 16], and the knowledge about the effect of hospitalisation and antimicrobial treatment on the faecal resistome is limited.

In the current study, we aimed to characterise the effects of hospitalisation and TMS administration on the faecal microbiota and resistome of healthy ponies with a follow-up period of 6 months to assess long-term effects and determine the potential relevance of the equine hindgut as a reservoir for ARGs.

## Materials and methods

### Study design

This longitudinal study was performed on six clinically healthy Welsh ponies (aged 10 to 11 years; all geldings) on a single farm and was carried out between February and September 2017. The ponies had no diet changes and no history of antimicrobial treatment for at least 7 years. All ponies were housed individually at the farm, received ad libitum hay, and were kept on pasture. The ponies were also housed individually and fed hay during hospitalisation, but had no pasture access. Behaviour and appetite were monitored daily, and rectal temperature was collected on sampling days. Faecal samples were collected at the following time points. Initial samples were collected at the farm on day 0 (D0) and again on day 13 (D13-1). Immediately after group transportation by horse truck (total duration 90 min) to Utrecht University Equine Hospital, the ponies were resampled (D13-2). The ponies were hospitalised without treatment for 7 days and sampled daily in the morning (D14–D21). At D21 (after collection of the D21 sample), oral treatment with trimethoprim-sulfadiazine (TMS) 5/25 mg/kg BID (Sulfatrim®, AST Farma, Oudewater, the Netherlands) was started for five consecutive days and samples were collected daily (D22 - D26; TMS treatment samples). On D27, the ponies were transported back to the farm as a group by horse truck and kept on individual pastures for the remainder of the study period (6 months). The ponies were again sampled immediately before and after transportation (D27-1, D27-2). The first week after discharge from the hospital, follow-up faecal samples were collected from the ponies daily (D28–D34) and subsequently weekly on D41, D48, and D58, after which the sampling continued monthly on D88, D119, D149, D180, and D211.

### Sampling

Faecal samples were collected rectally from individual ponies using a rectal glove. Four aliquots of 2 g per sample were stored at −80°C within 2 h of collection for subsequent microbiome and resistome analyses.

### DNA extraction, 16S rRNA gene sequencing, and shotgun metagenomic sequencing

DNA extraction was performed using the QIAamp Fast DNA Stool Mini Kit (Qiagen, Hilden, Germany) following a previously published protocol [17], with the minor modification of replacing the step of “treatment of samples in the TissueLyser at 30Hz for 3 × 30 s with cooling on ice in between treatments” by bead-beating the samples for 5 min on a Vortex-Genie 2 (Scientific Industries, Bohemia, New York, USA). For 16S rRNA gene sequencing, the variable V3 and V4 regions of the 16S rRNA gene were amplified (16S amplicon PCR forward primer = 5′ TCGTCGGCAGCGTCAGATGTGTATAAGAGACAGCCTACGGGNGGCWGCAG and 16S amplicon PCR reverse primer = 5′ GTCTCGTGGGCTCGGAGATGTGTATAAGAGACAGGACTACHVGGGTATCTAATCC), and libraries were prepared following the 16S rRNA gene Metagenomic Sequencing Library Preparation protocol (Illumina, San Diego, California, USA). Next, each library was normalised, pooled, and loaded onto the Illumina MiSeq platform for paired-end sequencing using the 600 cycles MiSeq Reagent Kit V3 (Illumina, San Diego, California, USA), generating 2 × 300 basepair paired-end reads. For shotgun metagenomic sequencing libraries were prepared with the Illumina Nextera XT kit. Multiplexing and sequencing were performed using the Illumina NextSeq platform (150 bp paired-end sequencing) targeted at 42 million reads per sample.

### Bioinformatics processing

Data preparation was performed using Jupyter notebook version 5.7.8, running on Python 3.7.3. utilising R version 3.4.4. To process the 16S rRNA gene sequencing data, raw reads (250 bp) obtained from Illumina 16S rRNA gene sequencing provided input for the denoising pipeline DADA2. DADA2 models and corrects Illumina-sequenced amplicon errors with high precision [18]. First, the forward and reverse reads were sorted, and the quality profile was plotted. Trimming parameters were derived from the quality plots, maintaining a minimum quality score of 20. Forward reads contained higher quality than reverse reads, common among Illumina data. Truncations were set at 15-290 for forward, and 15-210 for reverse reads. Post filter and trimming the reads were merged. Merged data was used to create a sequence table. Reads were grouped into amplicon sequence variants (ASVs). After removing chimaeras, taxonomy was assigned using v. 132 of the Silva database [19]. DNA reads from shotgun metagenomic sequencing were processed using FastDeME with default settings (https://github.com/aldertzomer/FastDeMe). Reads were processed using FastP to remove poor-quality reads and sequencing adapters and barcodes [20]. The tool KMA [21] was used to detect hits to known antimicrobial resistance genes with the default Resfinder database [22] to investigate the resistome. Bacterial, archaeal, and eukaryotic composition was determined by counting matching shotgun metagenomic reads to the 16S/18S SILVA database v.132 using Kraken2 [23]. Resistance gene abundances were transformed using additive log-ratio (ALR) by dividing by the total 16S rRNA gene counts per sample, times one hundred thousand for better readability and expressed on a log (ln) scale. The resistome was clustered at the AMR class and the 90% identity cluster level (ARG cluster, CD-HIT-EST) [24, 25]. Resistance classes were obtained from the Resfinder database [22], and hits to each resistance gene were summed per antimicrobial class.

### Data analysis

All data analysis and visualisation were performed with R version 4.0.2 [26] using Phyloseq [27], vegan [28], and ggplot [29] packages. All samples had at least 10,503 reads (mean 17,622 reads; median 16,192 reads, range 10,503–56,190 reads), and no samples were excluded.

### Microbiota composition

Relative abundances of bacterial taxa at the phylum level were assessed for each sample, and phylum-, class-, and for the major phyla, also family-level bar plots were produced. Statistical differences in relative abundance of taxa between samples collected at different time points during the study (D13-1 vs. D13-2; D13-1 vs. D21; D21 vs. D26 and D0 vs. D211) were computed (Wilcoxon signed rank), followed by adjustments for multiple testing (Benjamini-Hochberg procedure with alpha set at 0.1). Alpha-diversity (Shannon diversity) and observed richness (total number of species) were calculated from rarefied data. Data was rarefied to the sample with the lowest read counts (10,503 reads). Between-sample Bray-Curtis dissimilarity was computed on relative abundance data and used for non-metric dimensional scaling (NMDS). To determine if significant differences in microbiota composition (beta-diversity) were present between samples collected at different time points, permutational multivariate analysis of variance (PERMANOVA), including beta dispersion analysis, was performed (vegan package function Adonis2 and betadisper). For this analysis, samples were clustered in groups: start of study (D0–D13-1; *n*=12), hospitalisation without treatment (D14–D21; *n*=48), TMS treatment (D22–D26; *n*=30), short-term follow-up (D27–D34; *n*=48), and long-term follow-up (D41–D211; *n*=48). The samples collected immediately after transportation (D13-2 and D27-2) were excluded from this analysis to avoid interference of transportation effects. The permutation matrix was stratified per individual (“strata = horse”). Data were rarefied for random forest analysis, and the top 200 taxa were used. The analysis was performed with “start of study” and “TMS treatment” samples as classes and the microbial density data as classifiers using the “randomForest” package in Bioconductor [30]. The resulting random forest model was used to predict when microbiota composition was more similar to microbiota composition at the start of the study or during TMS treatment on the samples not used for training. To determine ASVs that differed in abundance between different time points, the DESeq2 package [31] was used. The DESeq2 package determines if a significant fold change is present with a Wald test. *P*-values were adjusted for the false discovery rate (Benjamini-Hochberg approach [32]) with alpha set at 0.01. Raw count data was used as input. ASVs seen more than three times in at least 20% of the samples were included. Percentages of interesting taxa were calculated to reveal abundances in the entire microbiome. Specific time points were chosen for comparison to assess the effect of the different interventions (transportation D13-1 vs. D13-2; hospitalisation D13-1 vs. D21; TMS treatment D21 vs. D26 and the cumulative effect of all interventions D0-13 vs. D180-211).

### Resistome

Shotgun metagenomic sequencing was performed on D13-1, D16, D20, D22, D24, D26, D28, D31, D34, D41, D58, D88, and D211 samples to study the resistome. The relative abundance of ARGs was calculated for each sample. The computed ALR data were visualised with bar plots at the antimicrobial class level. Statistical differences of ARGs between samples were computed (Wilcoxon signed rank), followed by adjustments for multiple testing (Benjamini-Hochberg procedure with alpha set at 0.1). The significantly different ARGs were visualised over time with scatterplots.

### Metagenomic assembly

The short reads obtained from shotgun metagenomic sequencing were assembled into longer contiguous sequence stretches (contigs), using SPAdes [33], to obtain information on the location of the ARGs in genomes present in the microbiome. Taxonomic classification of the assembled contigs was performed using CAT/BAT [34]. We aimed to identify which species of bacteria were harbouring the ARGs and if the identified ARGs were located on mobile genetic elements, such as plasmids.

## Results

All six ponies remained clinically healthy throughout the study period with no changes in rectal temperature, behaviour, and appetite (Additional file 1). After transportation to the hospital four ponies showed signs of excitation, mild sweating, or softer faecal consistency which resolved within 2 h after arrival.

### Oral TMS treatment disturbs the faecal microbiota composition and recovery is observed within 2 weeks after discontinuation of treatment to a large extent; however, significant differences could still be observed 6 months later

**Fig. 1:**
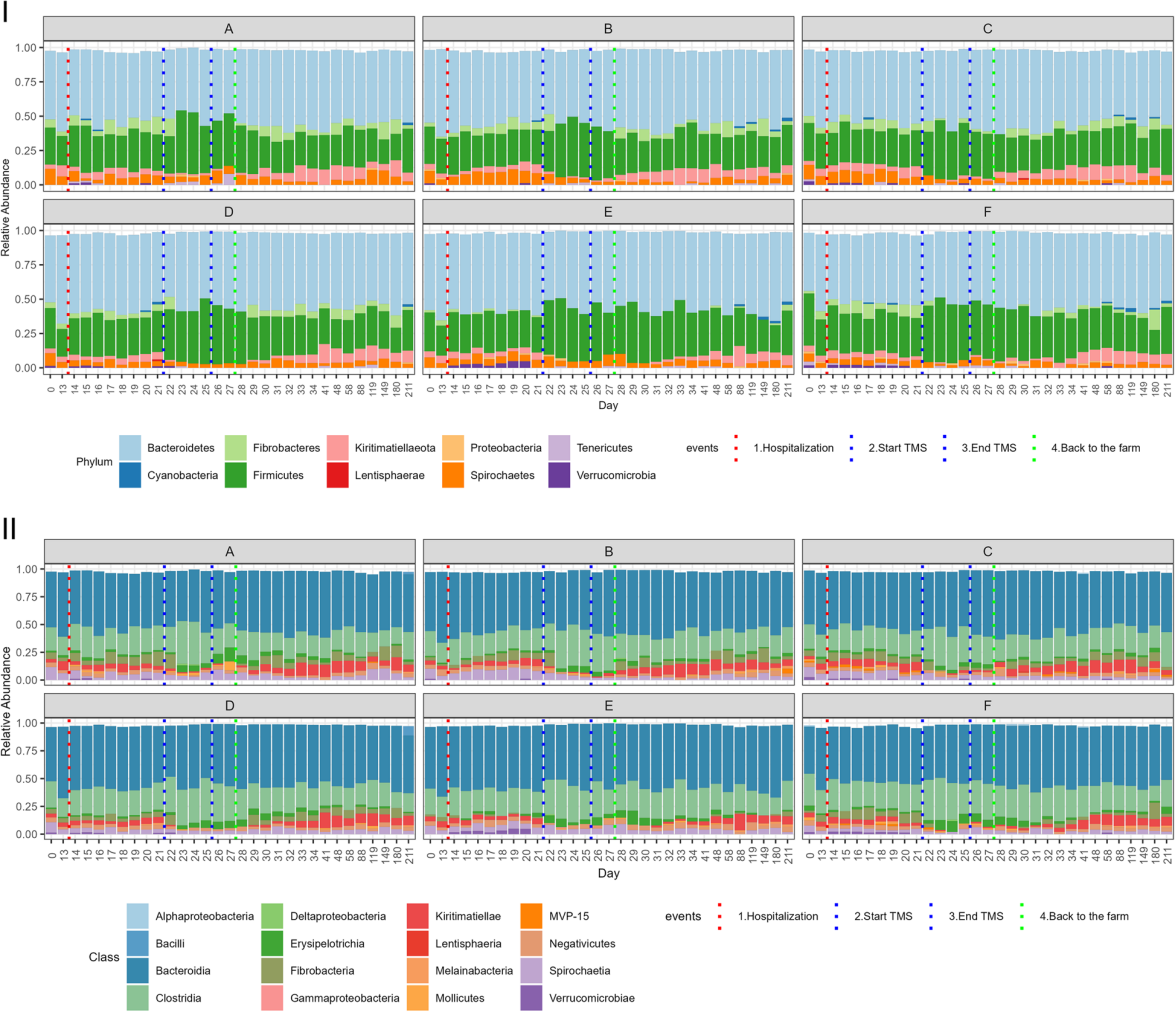
Relative abundance of the most abundant bacterial communities at (**I**) phylum and (**II**) class level in faecal samples collected from Welsh ponies (*A–F*) at the farm (D0–D13-1), during hospitalisation without treatment (D14–D21), during hospitalisation and treatment with TMS (D22–D26), and after discharge from the hospital up until 6 months after hospitalisation and antimicrobial treatment (D27–D211)

**Fig. 2:**
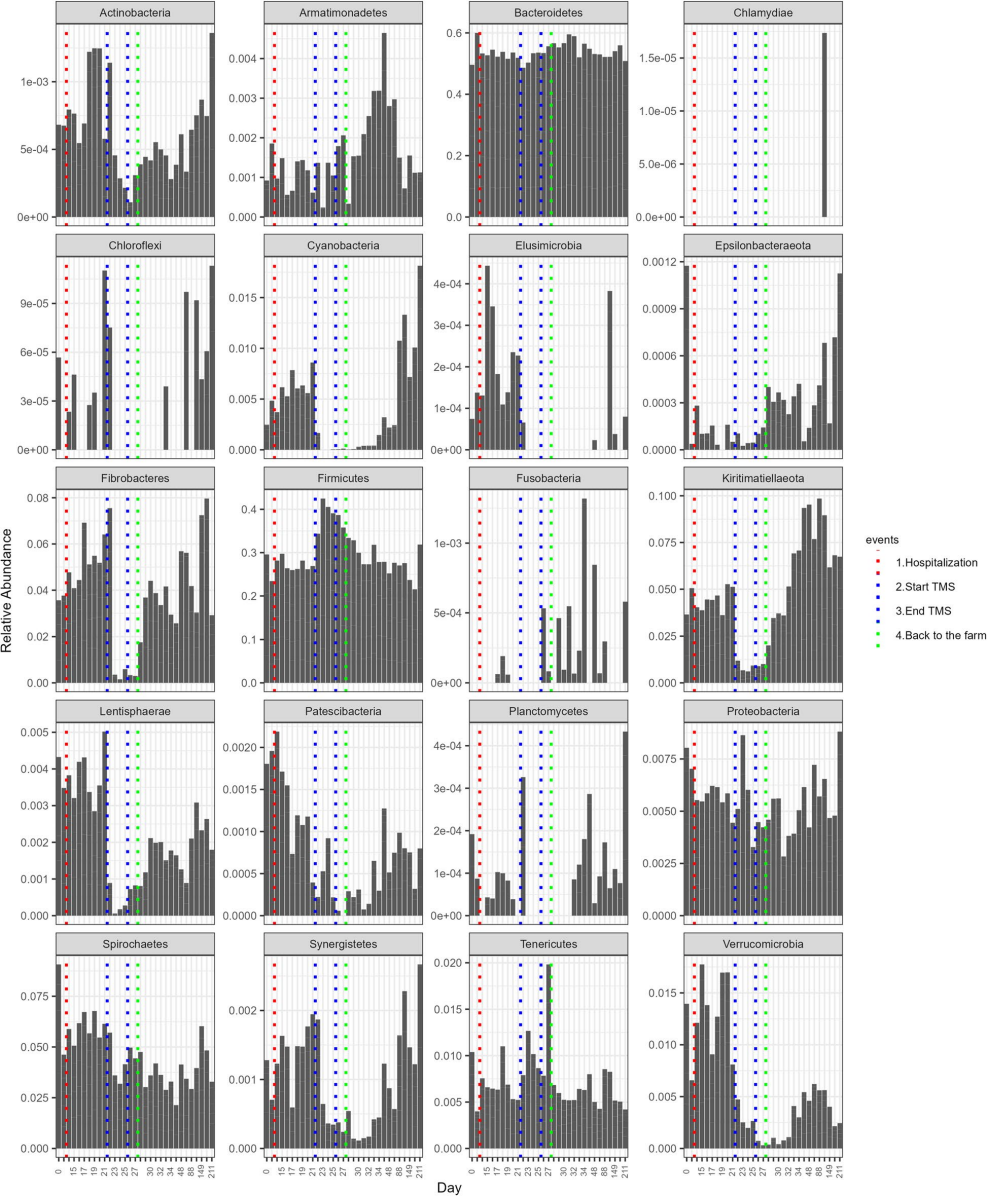
Mean relative abundance of specified phyla over time. Mean relative abundance of phyla in the faecal microbiota of Welsh ponies at the farm (D0–D13-1), during hospitalisation without treatment (D14–D21), during hospitalisation and treatment with TMS (D22–D26), and after discharge from the hospital up until 6 months after hospitalisation and antimicrobial treatment (D27–D211). Scaling on the *y*-axis varies according to relative abundance

**Fig. 3:**
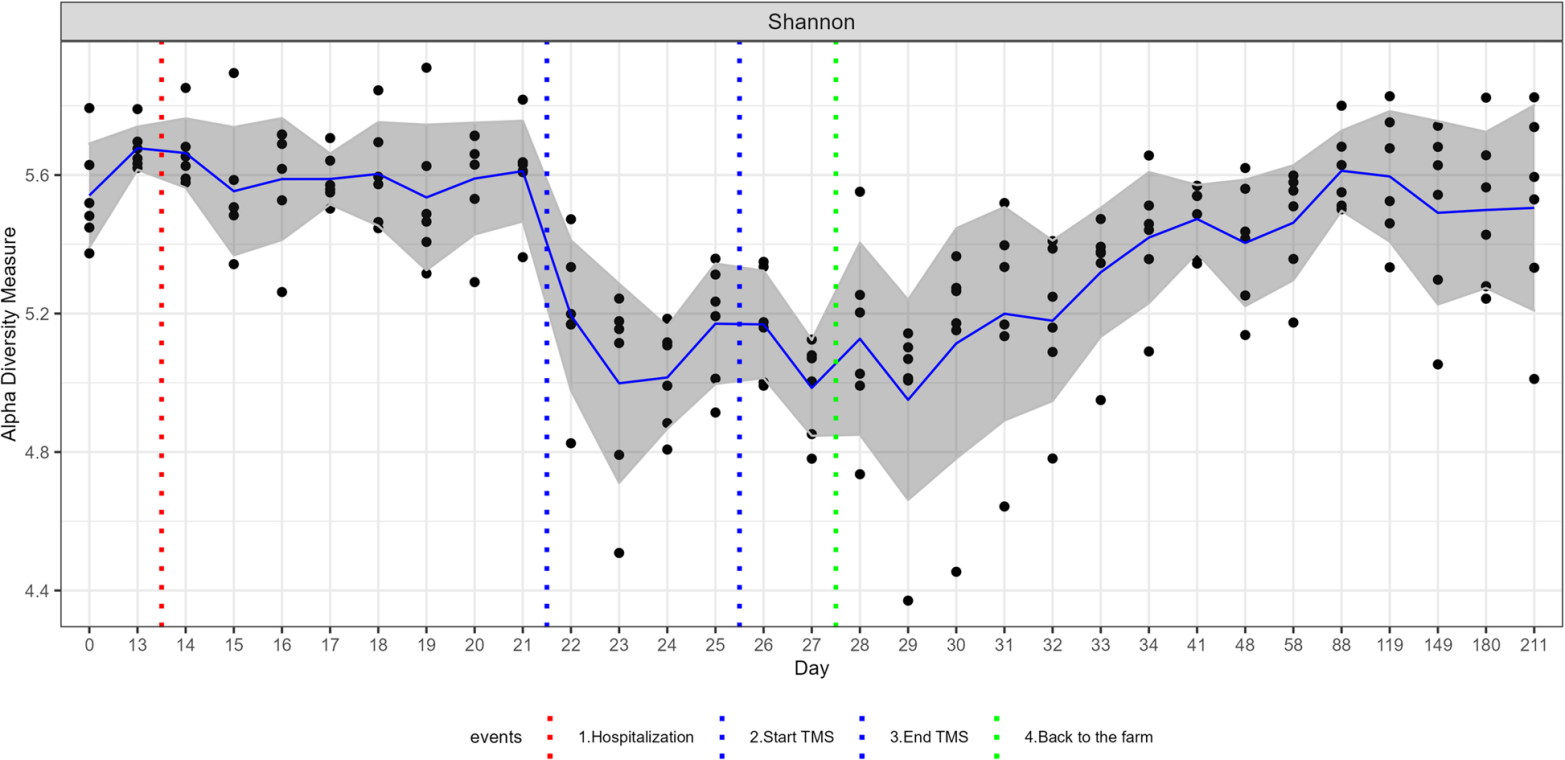
Alpha-diversity (Shannon) over time. Alpha-diversity of the faecal microbiota of Welsh ponies at the farm (D0–D13-1), during hospitalisation without treatment (D14–D21), during hospitalisation and treatment with TMS (D22–D26), and after discharge from the hospital up until 6 months after hospitalisation and antimicrobial treatment (D27–D211). The blue solid line is the mean, and the grey area represents one standard deviation

The composition of the faecal microbiota at phylum and class level and the mean relative abundance of the specified phyla over time are presented in Figs. 1 and 2. Relative abundance of specified phyla over time at the level of the individual ponies is presented in Additional file 2. Statistical differences in relative abundance of phyla between samples collected at different time points are presented in Additional file 3. For the two largest phyla, Bacteroidetes and Firmicutes, the relative abundance of families belonging to these phyla over time is presented in Additional file 4. At the start of the study (D=0), Bacteroidetes was the phylum with the largest relative abundance (49.5%), followed by Firmicutes (29.7%), Spirochaetes (8.9%), Kiritimatiellaeota (3.6%), Fibrobacteres (3.6%) and Verrucomicrobia (1.4%). All other phyla had a relative abundance of <1%. Mean alpha-diversity (Shannon) was 5.550, ranging from 5.394 to 5.799 (Fig. 3).

After transportation from the farm to the hospital (90 min duration), a sharp increase in alpha-diversity (Shannon) from 5.678 immediately before transport (D13-1) to 6.089 immediately after transport (D13-2; not shown) was observed. On the phylum level, an increased relative abundance of Firmicutes (23.5% vs. 28.5%; *p* 0.031) and a decreased relative abundance of Bacteroidetes (59.7% vs. 54.9%; *p* 0.031) and Synergistetes (0.12% vs. 0.08%; *p* 0.031) were observed; however, these were not significant after BH correction (Additional file 3). Differentially abundant ASVs grouped by family before and after transportation to the hospital (DESeq plots) are presented in Additional file 5. The effects of transportation on faecal microbiota composition were no longer present the day after hospital admission (D14).

**Fig. 4:**
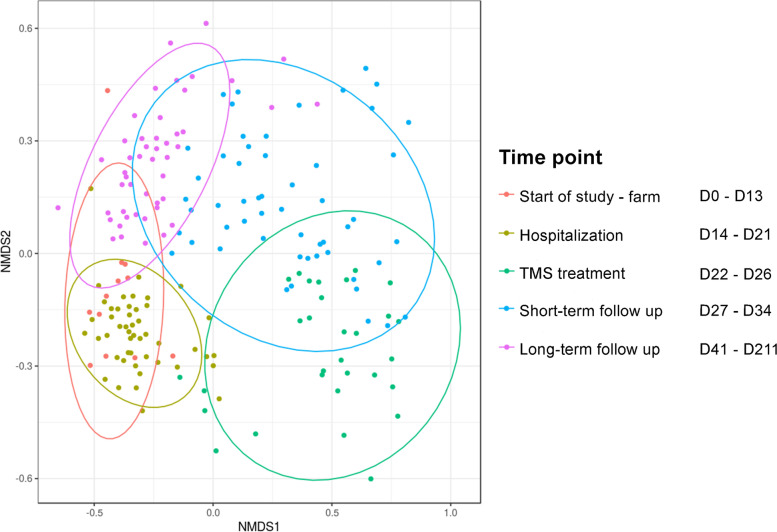
Compositional differences in faecal microbiota over time. Bray-Curtis non-metric multidimensional scaling (NMDS) plot of beta-diversity of the faecal microbiota of Welsh ponies at the farm (D0–D13-1), during hospitalisation without treatment (D14–D21), during hospitalisation and treatment with TMS (D22–D26) and after discharge from the hospital (short-term follow-up D27–D34) up until 6 months after hospitalisation and antimicrobial treatment (long-term follow-up D41–D211). Overall, PERMANOVA and pairwise PERMANOVA indicate significant differences between all groups (*p*<0.001), with six out of ten comparisons having significant differences in beta dispersion (*p*<0.05)

Hospitalisation without antimicrobial treatment for 7 days did not cause any significant changes in alpha-diversity (Fig. 3). On the phylum level, relative abundance of Bacteroidetes (59.7% vs. 51.9%; *p* 0.031) decreased and relative abundance of Synergistetes (0.08% vs. 0.20%; *p* 0.031) increased; however, these trends were not significant after BH correction (Figs. 1 and 2, Additional file 3). Also, no compositional differences in the faecal microbiota (beta-diversity) were observed (Fig. 4). However, some ASVs belonging to different bacterial families did differ significantly between D13-1 and D21 (Additional file 5).

Oral treatment with TMS for five consecutive days led to a significant decrease in alpha-diversity (Shannon) from 5.614 on D21 to 5.171 on D26 (Fig. 3). Relative abundance of several of the main phyla decreased rapidly after TMS treatment was started (Figs. 1 and 2): Kiritimatiellaeota (D21 5.7% vs. D26 0.9%; *p* 0.031), Fibrobacteres (D21 5.7% vs. D26 0.4%; *p* 0.031), and Verrucomicrobia (D21 0.7% vs. D26 0.1%; *p* 0.031). Also, the smaller phyla of Lentisphaerae and Cyanobacteria decreased significantly in relative abundance after TMS treatment. The relative abundance of the large phylum of Firmicutes significantly increased (D21 26.4% vs. D26 38.6%; *p* 0.031) due to TMS treatment. Within the phylum of Firmicutes, the increase in relative abundance was caused by an increase in the relative abundance of the bacterial families of *Ruminococcaceae*, *Erysipelotrichaceae*, and *Clostridiales_vadinBB60_group* (Additional files 4 and 5). Relative abundance of the bacterial family of *Veillonellaceae*, also belonging to the Firmicutes phylum, decreased after TMS treatment. The relative abundance of the large phylum of Bacteroidetes was unaffected; however, changes in the relative abundance of families belonging to this phylum were observed (Additional files 4 and 5). The bacterial families *Bacteroidales_UCG-001*, *Bacteroidales_BS11_gut_group*, *F082*, *Rikenellaceae*, *Bacteroidetes_BD2-2*, and *COB_p4-1_termite_group* all decreased in relative abundance, whereas the bacterial families *p-251-o5*, *Paludibacteraceae*, and *Prevotellaceae* increased in relative abundance. No changes in the relative abundance of Proteobacteria were observed. The observed trends were similar in all subjects. PERMANOVA indicates significant differences in beta-diversity before and after treatment with TMS (*p*<0.001), demonstrating clear compositional changes in the faecal microbiota (Fig. 4).

**Fig. 5:**
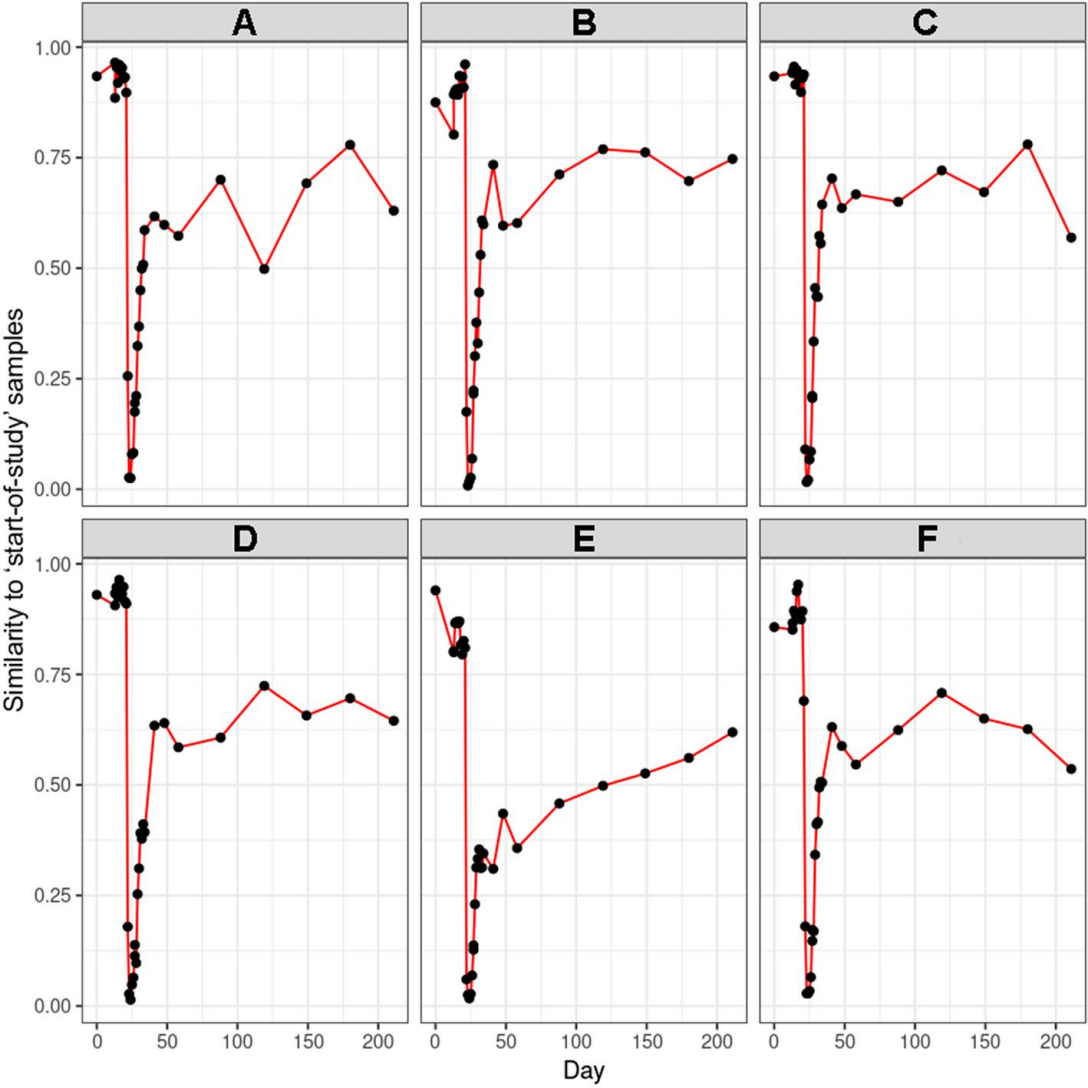
Random forest analysis of microbiota composition over time. Random forest analysis of faecal microbiota composition of Welsh ponies (**A–F**) at the farm (D0–D13-1), during hospitalisation without treatment (D14–D21), during hospitalisation and treatment with TMS (D22–D26), and after discharge from the hospital up until 6 months after hospitalisation and antimicrobial treatment (D27–D211). “Start of study” samples (D0-D13-1) and “TMS treatment” samples (D22–D26) were used as classes for training. 1 = 100% similarity with samples at the start of the study

No transport-related changes in faecal microbiota composition were observed when the ponies were discharged from the hospital and brought back to the farm. This is in contrast to the changes observed after transportation from the farm to the hospital 2 weeks earlier under identical conditions. After discharge and discontinuation of antimicrobial treatment, alpha-diversity (Shannon) increased again, approaching levels similar to that of the start of the study 2 weeks later (D0 5.550 vs. D41 5.470) (Fig. 3) and faecal microbiota composition was more similar to pre-treatment composition than to composition during TMS treatment at D41 for five out of six ponies, as can be observed in the random forest plots (Fig. 5). At D211, 6 months post-hospitalisation and TMS treatment, the mean alpha-diversity (Shannon) was 5.512, comparable to the mean alpha-diversity at the start of the study (D0 5.550) (Fig. 3). However, the relative abundance of some phyla did not fully recover and remained significantly lower in the 6-month follow-up period compared to the start of the study (Figs. 1 and 2). This was the case for Spirochaetes (D0 8.8% vs. D211 3.2%; *p* 0.031), Verrucomicrobia (D0 1.4% vs. D211 0.25%; *p* 0.031), and the smaller phyla of Lentisphaerae and Tenericutes. The relative abundance of other phyla significantly increased during the follow-up period compared to the start of the study. This was observed for the phyla Kiritimatiellaeota (D0 3.6% vs. D211 6.5%; p 0.031) and Cyanobacteria (D0–D13-1 0.25% vs. D211 1.84%; *p* 0.031). Looking at beta-diversity, a gradual and partial recovery of the faecal microbiota composition was observed. However, microbiota composition at D211 differed from that at the start of the study, as indicated in the NMDS plot (Fig. 4) and the random forest plots (Fig. 5). PERMANOVA between “start of the study” and “long-term follow-up” indicated significant differences between groups, as well as significant differences in beta dispersion. Differentially abundant ASVs grouped by family at the start of the study vs. 6 months after hospitalisation and TMS treatment (DESeq plots) are presented in Additional file 5.

### Oral treatment with TMS has a rapid and long-lasting effect on the faecal resistome

**Fig. 6:**
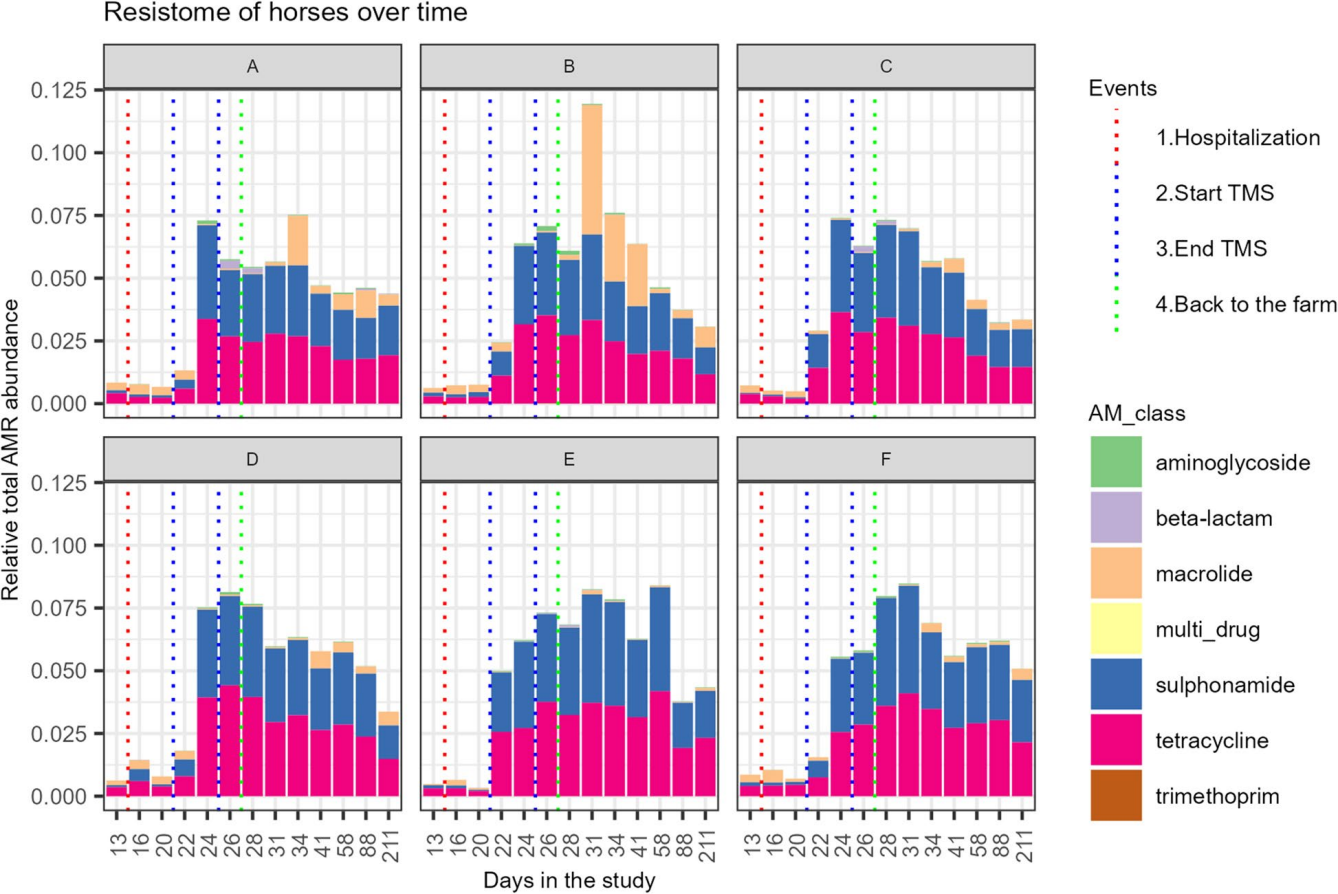
Resistome abundance in the faecal microbiome. Stacked bar chart of the relative abundance of resistance genes clustered at the antimicrobial class level observed in the faecal microbiome of Welsh ponies (**A–F**) at the farm (D0–D13-1), during hospitalisation without treatment (D14–D21), during hospitalisation and treatment with TMS (D22–D26), and after discharge from the hospital up until 6 months after hospitalisation and antimicrobial treatment (D27–D211)

**Fig. 7:**
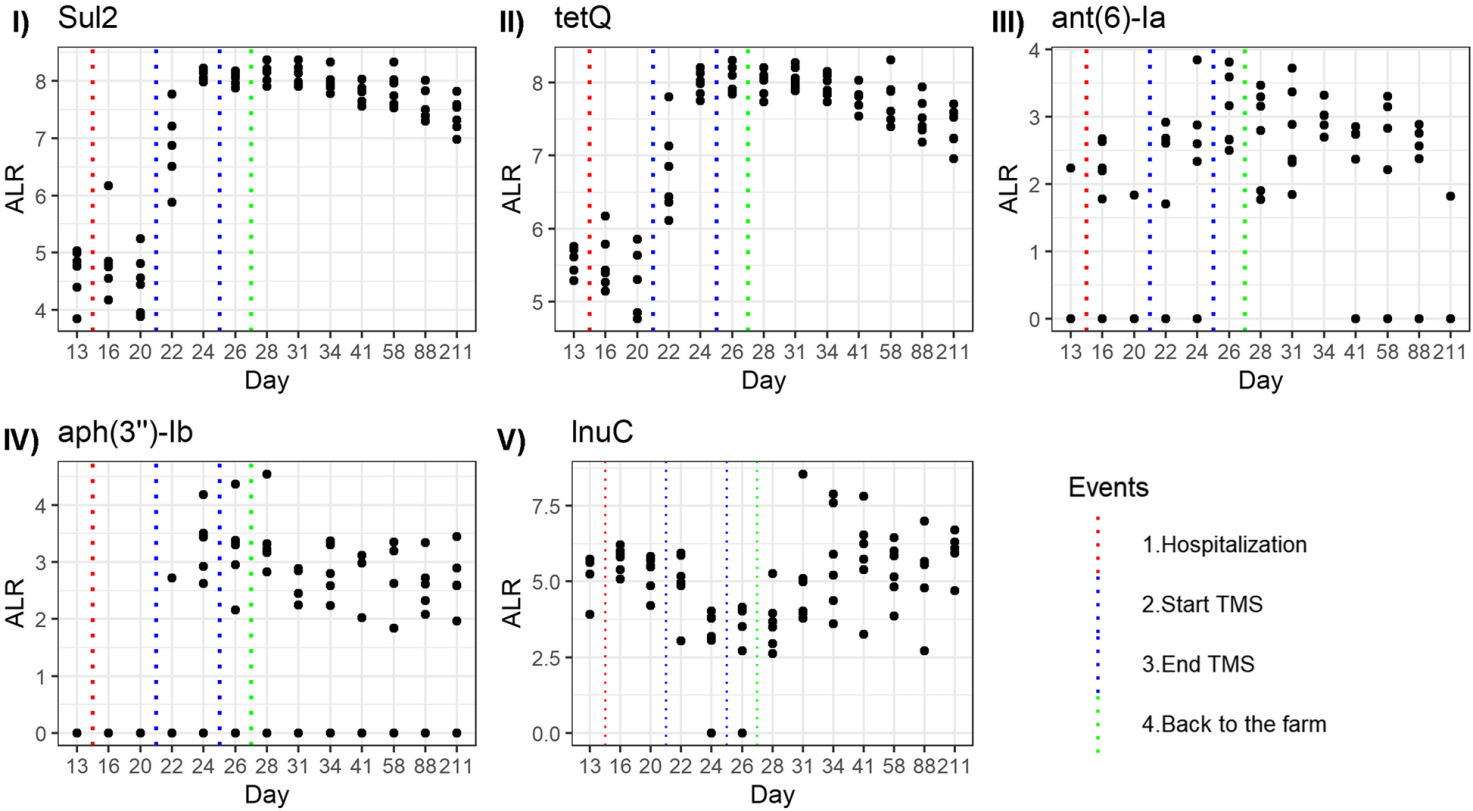
Relative abundance of resistance genes over time. Relative abundance of resistance genes in the faeces of Welsh ponies at the farm (D0–D13-1), during hospitalisation without treatment (D14–D21), during hospitalisation and treatment with TMS (D22–D26), and after discharge from the hospital up until 6 months after hospitalisation and antimicrobial treatment (long-term D27–D211). **I** sul2, **II** tetQ, **III** ant(6)-la,** IV** aph(3”)-lb, **V** lnuC. ALR: additive log-ratio

The relative abundance of resistance genes in the faecal microbiome in all study subjects is presented in Fig. 6. At the start of the study, the relative abundance of resistance genes was low. Resistance genes *lnuC* (lincomycin resistance), *sul2* (sulfonamide resistance), *tet40*, *tetQ*, and *tetW* (tetracycline resistance) were detected in faecal samples from all six ponies. Resistance genes *ant(6)-la* (aminoglycoside resistance), *mef(A)* (macrolide resistance), and *tet(O/32/O)* (tetracycline resistance) were identified in faecal samples from some, but not all, ponies. Hospitalisation without antimicrobial treatment for 7 days did not significantly affect the relative abundance of resistance genes in the faeces of the ponies included in this study (Figs. 6 and 7).

After oral TMS treatment, a significant increase in the relative abundance of several resistance genes was observed (D20 vs D26): a 32-fold increase in sulphonamide resistance gene *sul2* and a 16-fold increase in tetracycline resistance gene *tetQ* (Figs. 6 and 7). Aminoglycoside resistance genes *ant6-1a* and *aph(3”)-lb* also increased in all subjects after treatment with TMS, but these genes were below the detection limit prior to treatment in most subjects, hindering the calculation of a fold increase. The relative abundance of lincomycin resistance gene *lnuC* significantly decreased six-fold directly after treatment. Differences between individual ponies were present, demonstrated by the relatively large increase in macrolide resistance genes in ponies A and B, whereas this increase was limited in ponies C, D, E, and F.

Six months after antimicrobial treatment, *sul2* (15-fold) and *tetQ* (six-fold) resistance genes remained significantly increased at D211 compared to D0 (Figs. 6 and 7). Three new types of ARGs were observed in the faeces of some subjects at D211, which were not detected at the start of the study: *aph(3”)-lb*, *blaACI* and *cfxA6*.

By assembling the short reads into longer contigs, we aimed to identify which species of bacteria were harbouring the ARGs and evaluate if the observed ARGs were located on mobile genetic elements, such as plasmids. We observed that resistance genes *sul2* and *tet(Q)* were located on the same contig observed in the genome of an unidentified bacterial species belonging to the class of Bacteroidales (phylum Bacteroidetes). Furthermore, resistance genes *aph(3”)-lb* and *aph(6)-ld* (and sometimes also *strA_1*) were located on the same contig observed in the genome of a bacterial species belonging to the genus of *Desulvibrio* (phylum Proteobacteria).

## Discussion

Faecal microbiota composition at the phylum level was similar to previously reported in healthy equids from the same geographic area [2] and other geographic areas [35–37]. Individual variation among the ponies included in our study was limited, potentially as a result of the fact that these ponies were housed under the same circumstances and fed the same diet for > 7 years. Bacteroidetes was the phylum with the largest relative abundance, followed by Firmicutes. However, in other studies, Firmicutes are reported to be the largest phylum [38–42]. These large differences in microbiota composition between studies might result from technical differences in protocols for DNA extraction, sequencing and data analyses, limiting the comparison of results from different studies.

We noticed a sharp increase in alpha-diversity and richness of the faecal microbiota directly after transport to the hospital compared to samples collected 90 min earlier, prior to transportation. We also observed shifts in the relative abundance of major phyla, such as Firmicutes and Bacteroidetes. These changes were of short duration and were no longer observed in samples collected the following day. This contrasts with a previously published study reporting no differences in alpha-diversity and relative abundance of phyla after transportation [42] and another study describing changes in faecal microbiota only to be observed 3 days later [15]. Transportation can cause significant stress in horses [43]. A study in rats showed that stress significantly increased gastrointestinal transit time [44]. The ponies in the current study were not used to transportation and may have experienced stress. Three ponies showed signs of stress (excitation or mild sweating) after transportation, and soft faeces (but no diarrhoea) was observed in four out of six ponies. Stress might have led to increased gastrointestinal transit time and could potentially have caused the changes observed in faecal microbiota composition. Interestingly, we did not observe any changes when the ponies were transported back to the farm under identical circumstances. The ponies might have been more accustomed to new situations and less susceptible to stress after their 2-week stay at the hospital. Also, the diet prior to the two moments of transportation was different (mainly pasture vs. hay only) which might have an effect on the stability of the faecal microbiota composition. We can conclude that transportation can have a significant short-term effect on faecal microbiota composition, and this should be considered when designing future microbiota studies in horses.

In the current study, we did not observe a significant effect of hospitalisation without treatment on microbiota composition, but this might result from the small number of animals making it difficult to observe subtle changes. To our knowledge, no next-generation sequencing studies have been performed on horses to evaluate the effect of hospitalisation without antimicrobial treatment on faecal microbiota composition.

Generally, in all animals, irrespective of the type of antimicrobial administered, a decrease in species richness and alpha-diversity is observed [45], as was also the case in our study. The most profound effects of TMS on the intestinal microbiota were observed immediately after treatment, which is in line with previous reports that also demonstrated a rapid, significant decrease in alpha-diversity [5, 35, 46]. Relative abundance of Kiritimatiellaeota, Fibrobacteres, and Verrucomicrobia decreased significantly, suggesting that TMS has a strong action against bacteria in these phyla. As a result, a relative increase in the abundance of taxa belonging to Firmicutes was observed after TMS treatment. The decrease of Fibrobacteres (degradation of plant-based cellulose [47]) and Verrucomicrobia (mucin degradation [48]) are of interest as they are thought to have a positive effect on equine intestinal health [49, 50]. The observed trends were similar in all study subjects, which corresponds to another study reporting the effect of TMS on the equine faecal microbiota to be predictive [5] but contrast with a recent study reporting high inter-individual variability [35]. In that last study, the authors did not specify horse characteristics such as age, gender, and breed, which might affect response to treatment [35]. Also, the horses in that study had not received antimicrobials for only 3 months prior to the study, so potential previous antimicrobial administration might still have affected microbiota composition [35]. However, by using long-read sequencing, the authors of that study observed that species *Phascolarctobacterium* (phylum Firmicutes) and *Subdivision 5* (former phylum Verrucomicrobia, currently Kiritimatiellaeota [51]) decreased after TMS administration, whereas *Paraprevotella* (phylum Bacteroidetes) increased [35]. Due to the use of short-read sequencing in the current study, we could not detect changes in relative abundance at the species level, limiting direct comparisons between the two studies. However, we also observed a significant decrease in taxa belonging to the phylum Kiritimatiellaeota, anaerobic saccharolytic bacteria found in the mucous layer of the intestine of horses [51]. The same decrease has also been observed in another study assessing the effect of TMS on the faecal microbiota [5]. In that study, they also observed that the relative abundance of Firmicutes increased in response to TMS treatment, similar to what we observed in our study [5].

TMS has a more pronounced effect on the faecal microbiota than other antimicrobials, such as ceftiofur and penicillin [5]. This might result from the administration route (oral vs. parenteral). In a study in swine, oral oxytetracycline resulted in more pronounced changes in the faecal microbial community and a higher relative abundance of ARGs than parenteral injection with the same drug [52]. Future studies comparing oral versus intravenous administration of TMS in horses could provide more insight into the effect of the route of administration on microbiota and resistome composition.

Both random forest analysis and alpha-diversity analysis show that roughly 2 weeks after cessation of TMS treatment and discharge from the hospital (D41), microbiota composition was more similar to pre-treatment composition than composition during TMS treatment and thus indicates partial recovery of faecal microbiota composition. However, significant differences in beta-diversity could still be observed, indicating that recovery of microbiota composition is incomplete. In another study assessing the effect of oral TMS on faecal microbiota composition, the authors also observed recovery of microbiota composition to a large extent on day 25 after TMS treatment [5]. In that study, some differences in faecal microbiota composition were still evident 25 days after treatment, but no additional follow-up samples were collected to assess long-term effects. In the current study, the relative abundance of several main phyla, such as Spirochaetes, Fibrobacteres, and Verrucomicrobia, was still lower 2 weeks after discharge from the hospital compared to pre-treatment abundance. Kiritimatiellaeota increased at D41 (4.4% vs. 9.3%), indicating rebound “overgrowth” in the absence of TMS after the microbiota was significantly disturbed. The same trend was observed for some less dominant phyla, such as Cyanobacteria, Armatimonadetes, Epsilonbacteraeota, and Fusobacteria, indicating microbiota recovery after antimicrobial treatment is a slow and dynamic process. Six months after discontinuation of TMS treatment and discharge from the hospital (D211), significant differences in faecal microbiota composition could still be observed. The relative abundance of Spirochaetes, Verrucomicrobia, and the less abundant phyla of Lentisphaerae and Tenericutes was still lower than in start-of-study samples. In contrast, the relative abundance of Kiritimatiellaeota and Cyanobacteria was higher 6 months after hospitalisation and TMS treatment. Subtle yet long-lasting (up to 180 days) effects of antimicrobial exposure were also observed in a study on human subjects receiving a cocktail of meropenem, gentamicin, and vancomycin [53]. Long-lasting changes in microbiota composition in horses might be relevant as, for example, a decrease in Verrucomicrobia has been associated with diarrhoea in horses [4]. We can conclude that from the moment TMS treatment was stopped and the ponies were discharged from the hospital, a gradual recovery of faecal microbiota composition could be observed. However, microbiota composition never completely returned to pre-treatment composition in the study period.

At the start of the study, eight different ARGs could be detected in the faeces of our study subjects. Only the resistance genes *lnuC*, *sul2*, *tet40*, *tetQ*, and *tetW* were present in the faeces of all six ponies. Resistance genes *ant(6)-la*, *mefA*, and *tetO* were identified in some, but not all, ponies. These findings correspond with a recent study describing ARGs in faeces of healthy foals less than 30 days of age in Australia, in which *tetQ*, *tetO*, and *tetW* were most abundant, followed by *aphA3*, *sat4*, and *mefA* [16]. Studies of the resistome of pigs and poultry in intensive livestock farming systems [25] and healthy humans [54] seem to indicate a much higher number of different resistance genes. However, comparisons are difficult due to large differences in sample size, geographic location and housing conditions, and interaction with the environment and other individuals. Nevertheless, similar to our results in equids, tetracycline resistance genes were most abundant in human and pig faeces [25, 54]. In the current study, the animals were housed extensively and had no contact with other animals (although the pasture was injected with cow manure 12 months prior to the study), limiting their exposure to ARGs from their environment. They remained in their own closed ecological compartment, potentially explaining the low numbers of ARGs present in the faeces at the start of the study. This situation differs significantly from animals kept under intensive livestock farming conditions in which large numbers of animals are kept in close contact. Human individuals also do not live in a closed ecological compartment as they interact with their environment and other humans and animals, most likely leading to higher exposure to ARGs and, therefore, a more extensive resistome. Geographic location also affects the resistome. When the faecal resistome from humans, pigs, and poultry from different countries were compared, clear geographic differences in prevalence of ARGs were observed [25, 54]. Subjects from Denmark, a country with a restrictive antimicrobial use policy, showed the lowest ARG levels in both animals and humans. The low prevalence of ARGs in equine faecal samples collected at the start of the current study in the Netherlands could also be a geographically restricted finding, as a result of the strict antimicrobial use policy in the Netherlands. This should be considered when extrapolating this information to other equine populations.

In the current study, we did not observe an effect of hospitalisation without treatment on the faecal resistome. Implementation of an effective infection prevention protocol in the equine hospital in the current study might have prevented or reduced the dissemination of antimicrobial-resistant bacteria and ARGs to hospitalised horses. However, previous studies using culture-based or PCR techniques have shown that antimicrobial-resistant *E. coli* bacteria increase in the faeces of horses, independent of antimicrobial administration, within days of hospitalisation [55–60]. One study compared the duration of faecal shedding of resistant *E. coli* after antimicrobial treatment between hospitalised horses and non-hospitalised horses [61]. Two weeks following antimicrobial treatment, the odds of detecting resistant isolates in faeces of hospitalised horses were still increased. In contrast, in the non-hospitalised group, detection of resistant isolated returned to pre-treatment levels, suggesting that hospitalisation in that study affected the persistence of ARGs in the faeces after antimicrobial treatment. Culture and PCR techniques, as used in the studies described above, are very sensitive in detecting target isolates and resistance genes; however, no information is collected on other bacteria and ARGs than the targeted ones in the gut microbiota. Shotgun metagenomic sequencing, as used in the current study, does provide information on all bacteria and their full genetic potential (including ARGs) in the microbiota. However, it is less sensitive to detect subtle changes in the prevalence of ARGs due to limitations in sequencing depth. This might, in part, also explain the differences in results. To evaluate the actual effect of hospitalisation on the (duration of) faecal shedding of ARGs, more extensive shotgun metagenomic sequencing studies, including a non-hospitalised control group, are warranted.

Under antimicrobial selection pressure, only ARG-containing bacteria can grow and colonise the gut. The relative abundance of ARGs present at the start of the study before TMS treatment did increase after TMS treatment (enrichment of the intrinsic resistome). We also detected new ARGs in the faeces after antimicrobial treatment that were absent or below the limit of detection at the start of the study, indicating an increase in the diversity of the faecal resistome. A significant increase in the relative abundance of several ARGs could be observed within 24h after treatment with TMS. *Sul2*, *tetQ*, *ant6-la*, and *aph(3”)-lb* all increased in response to TMS treatment. Unfortunately, we could not observe trimethoprim resistance, as this is generally caused by a point mutation in the *dfr* gene, and the number of *dfr* genes in the databases is minimal. The rapid increase in ARGs shows that selection for (bacteria containing) ARGs occurs even after only 1 day of treatment. Antimicrobial resistance gene *lnuC* decreased in relative abundance after TMS treatment, indicating that this gene was most likely present in bacteria susceptible to TMS. Up until now, only one study using shotgun metagenomic sequencing has been performed to evaluate the effect of antimicrobial treatment on the excretion of ARGs in faeces of horses [12]. This study demonstrated an increase in the faecal presence of ARGs to several antimicrobial classes (tetracyclines, phenicol, macrolides, glycopeptides, aminoglycosides, and bacitracin) in foals from farms with endemic *Rhodococcus equi* infections after oral treatment with a combination of a macrolide and rifampin. Similar to our findings, ARGs encoding for resistance to other classes of antimicrobials than the drugs used for treatment increased significantly. This might be explained by co-selection due to the presence of multiple ARGs on mobile genetic elements, such as plasmids. This is supported by our finding that sulphonamide and tetracycline resistance genes *sul2* and *tetQ* were located on the same contig as well as aminoglycoside resistance genes *aph(3”)-lb* and *aph(6)-ld* (and sometimes also *strA_1*). Many ARGs seem to be located on mobile genetic elements, demonstrating the high risk of horizontal gene transfer between bacteria. This provides a cautionary example of the potential consequences of (injudicious) use of antimicrobials in horses.

The relative abundance of ARGs decreased slowly after TMS treatment was stopped, and the ponies were discharged from the hospital. Using culture-dependent techniques, it has been demonstrated before that administration of antimicrobials in horses increases resistant bacteria in the faeces for at least 2 weeks, but it is unknown how long these effects last [61, 62]. In cattle, subtherapeutic concentrations of antimicrobials led to higher faecal excretion of sulphonamide-, tetracycline-, and erythromycin resistance genes, and for *sul1*, excretion levels remained increased for 175 days [63]. In contrast, in a small study involving human subjects, 6 months post antimicrobial treatment, no significant differences in the relative abundance of resistance genes were observed [53]. In our study in equids, 6 months after hospitalisation and TMS treatment (D211), two ARGs (*sul2* and *tetQ*) were still detected in significantly higher numbers than pre-treatment, whereas faecal microbiota composition returned to pre-treatment composition to a large extent. Therefore, we can conclude that ARGs are present in a larger proportion of the microbiota, either in more species of bacteria, as a result of horizontal gene transfer between different species or by a shift from susceptible to resistant strains within bacterial species that persist. The former has been demonstrated in a study in mice in which a plasmid conferring multidrug resistance transferred from pathogenic *Salmonella enterica* isolates to commensal *E. coli* isolates in the gut microbiota [64]. The latter was recently observed in the previously mentioned study in foals treated for *Rhodococcus equi* infection, in which the relative abundance of a non-target organism, *Enterococcus* spp., did not change after antimicrobial treatment. However, the proportion of resistant isolates increased significantly [12]. Long-read and meta3C sequencing [65], a high-throughput chromosome conformation capture technique that allows complete resolution and linking genomes and plasmids in a mix of organisms, would be needed to assess to what level the processes mentioned above contributed to our findings in the equine gut.

The prolonged significant increase in ARGs in equine faeces after antimicrobial treatment demonstrated in this study highlights the potential relevance of the equine hindgut from a One Health perspective. Spread of ARGs between ecological compartments might be further facilitated by the rich hindgut microbiome of horses, the close contact between horses and humans, the overlap in microbiome components between horses and humans, the agricultural use of equine manure, and the fact that many antimicrobials used in horses are also used in human medicine.

While consistent with many other equine microbiome studies, the number of subjects included in the current study was low, which may have limited our ability to detect subtle differences in faecal microbiota composition and relative abundance of ARGs through limitations in statistical power. However, the effect of oral TMS on the faecal microbiota is substantial, and all subjects showed consistent trends in the changes observed, allowing us to draw conclusions based on a limited number of ponies. Also, given the longitudinal study design, and by including sampling before the interventions, the ponies served as their own controls. It is possible that the three successive interventions (transportation, hospitalisation, and antimicrobial treatment with TMS) affected one another, limiting our ability to study the effect of each intervention separately. However, in a clinical situation, these interventions also often occur in the same sequence, justifying studying the cumulative “real-world” effect. Our subjects were limited to one geographic region, dictating caution when extrapolating these results to other equine populations. In the current study, we used healthy ponies on a stable diet without antimicrobial exposure for >7 years. It remains to be determined if the same trends will be observed in (critically) ill patients treated in a hospital setting, especially those treated for gastrointestinal diseases. Due to the limited availability of *dfr* genes in the ARGs database, trimethoprim resistance was not identified and included in our analysis. Lastly, the assembly of metagenomic data in our study was challenging as multiple genomes in varying levels of abundance were present in each sample. Also, many of the bacteria in the equine gut microbiome belong to currently unidentified taxonomic lineages, limiting the ability to identify individual bacterial species that harbour ARGs. A follow-up study using long-read and meta3C sequencing would possibly produce more detailed information.

## Conclusions

Successive transportation, hospitalisation, and oral TMS treatment led to large and consistent changes in the equine faecal microbiota and a rapid and significant increase in the relative abundance of several ARGs. A gradual, relatively fast, but incomplete recovery of the faecal microbiota composition was observed after cessation of treatment and discharge from the hospital. However, the relative abundance of ARGs decreased very slowly and did not return to pre-treatment levels 6 months later. The prolonged significant increase in ARGs in equine faeces after antimicrobial treatment demonstrated in this study highlights the potential relevance of the equine hindgut from a One Health perspective. Therefore, judicious use of antimicrobials in horses is warranted to prevent the spread of resistance genes in the environment and minimise the potential dissemination of antimicrobial-resistant bacteria from horses to humans.

## Supplementary Information

**Additional file 1.** Data regarding appetite, behaviour and rectal temperature for all subjects during the entire study period.

**Additional file 2.** Relative abundance of specified phyla over time at the level of the individual ponies.

**Additional file 3.** Statistical differences (Wilcoxon signed rank) in relative abundance of taxa between samples collected at different time points during the study (D13-1 vs. D13-2; D13-1 vs. D21; D21 vs. D26 and D0 vs. D211).

**Additional file 4.** Relative abundance of bacterial families of the Bacteroidetes and Firmicutes phylum in faecal samples collected from Welsh ponies at the farm, during hospitalisation without treatment, during hospitalisation and treatment with TMS and after discharge from the hospital with six months follow-up.

**Additional file 5.** Differentially abundant ASVs grouped by family at different time points during the study (before vs. after transportation; before vs. after hospitalisation for one week without antimicrobial treatment; before vs. after five days of treatment with TMS; the start vs. the end of the study).

## Data Availability

The datasets supporting this article are available in the Sequence Read Archive repository as part of the supplementary electronic material and are available under accession PRJEB52712 (https://www.ncbi.nlm.nih.gov/bioproject/PRJEB52712/).

## Abbreviations

ALR: Additive log-ratio
ARGs: Antimicrobial resistance genes
ASVs: Amplicon sequence variants
TMS: Trimethoprim-sulfadiazine
