# Supplementary material for: Longitudinal study of the short- and long-term effects of hospitalisation and oral trimethoprim-sulfadiazine administration on the equine faecal microbiome and resistome
Source: Microbiome. 2023 Feb 27;11:33. doi: 10.1186/s40168-023-01465-6 (PMC9969626; doi:10.1186/s40168-023-01465-6)
Supplement: Supplementary file 3 — Additional file 2. Relative abundance of specified phyla over time at the level of the individual ponies. [file 40168_2023_1465_MOESM2_ESM.docx]

**Additional file 2.**

**Relative abundance of specified phyla at the level of the individual pony over time*.***

**A**

*
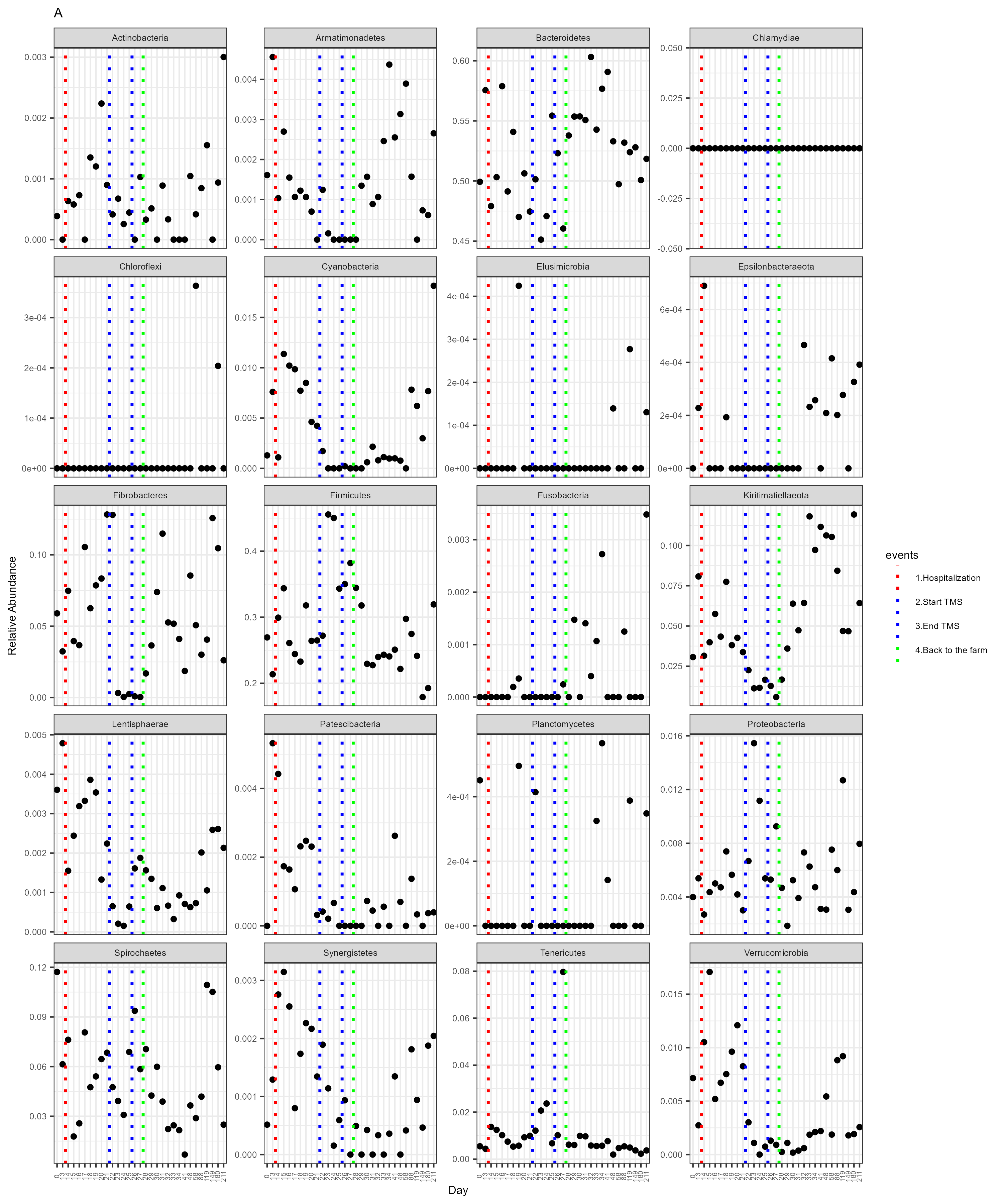
*

**B**

**
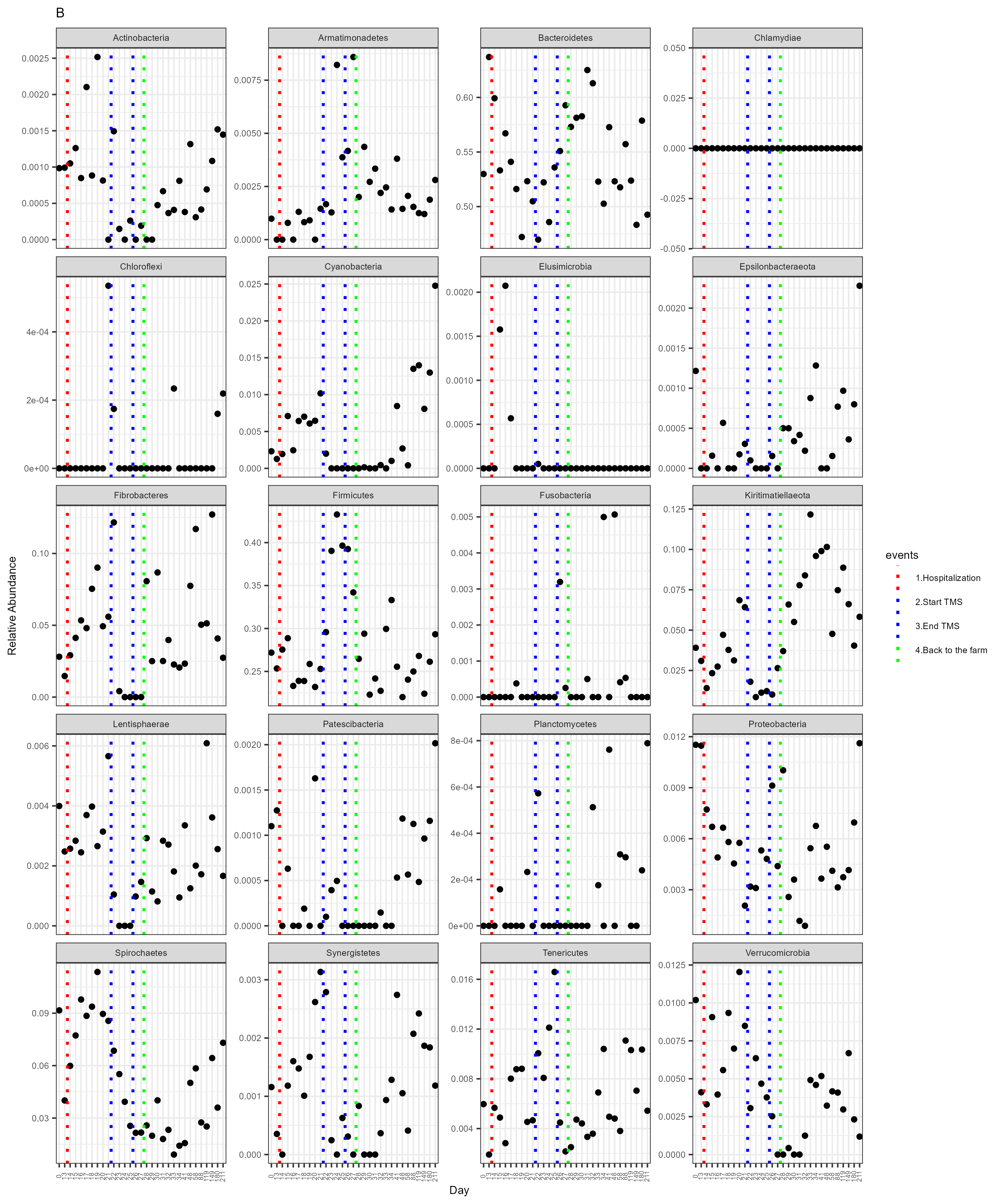
**

**C**

**
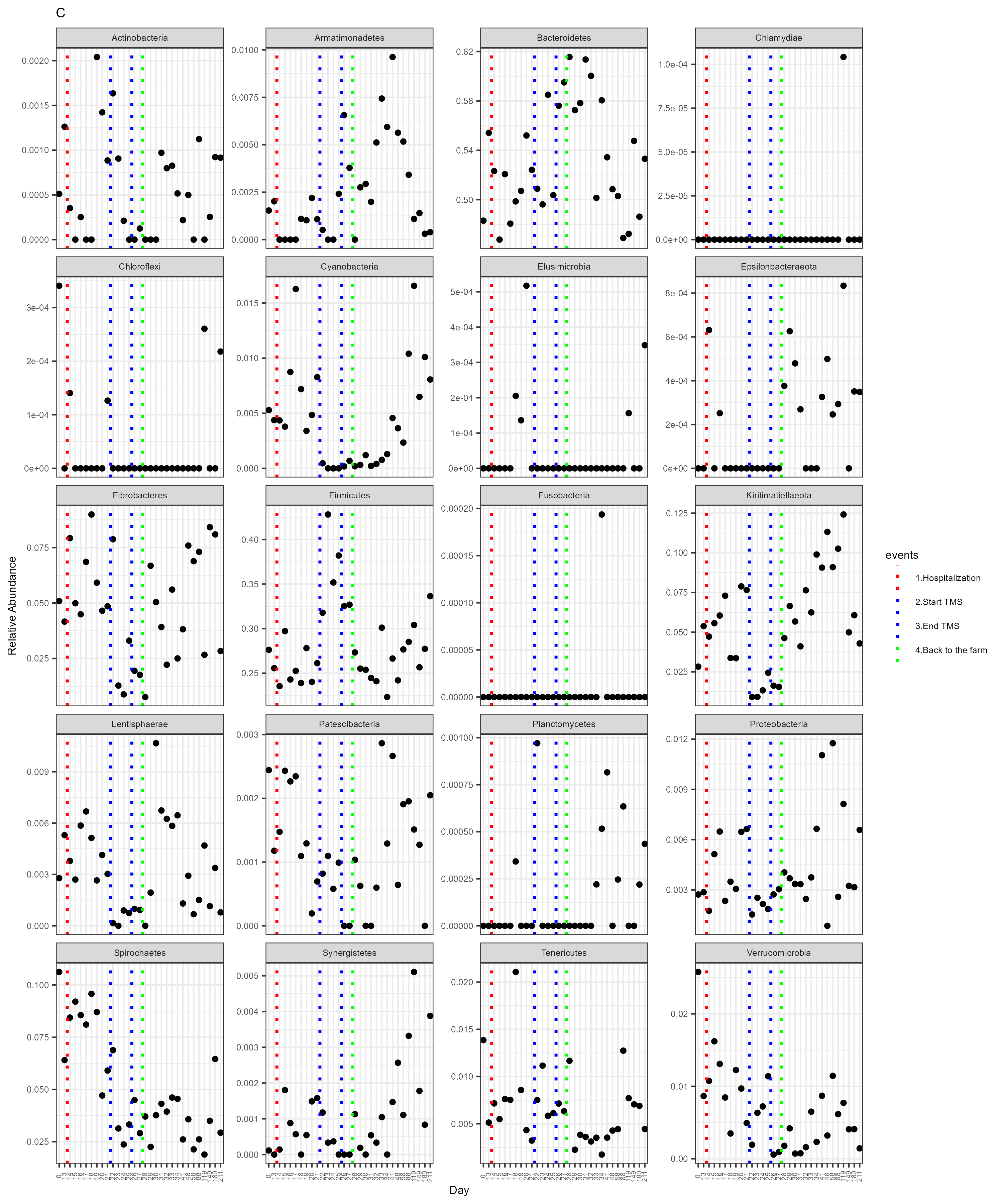
**

**D
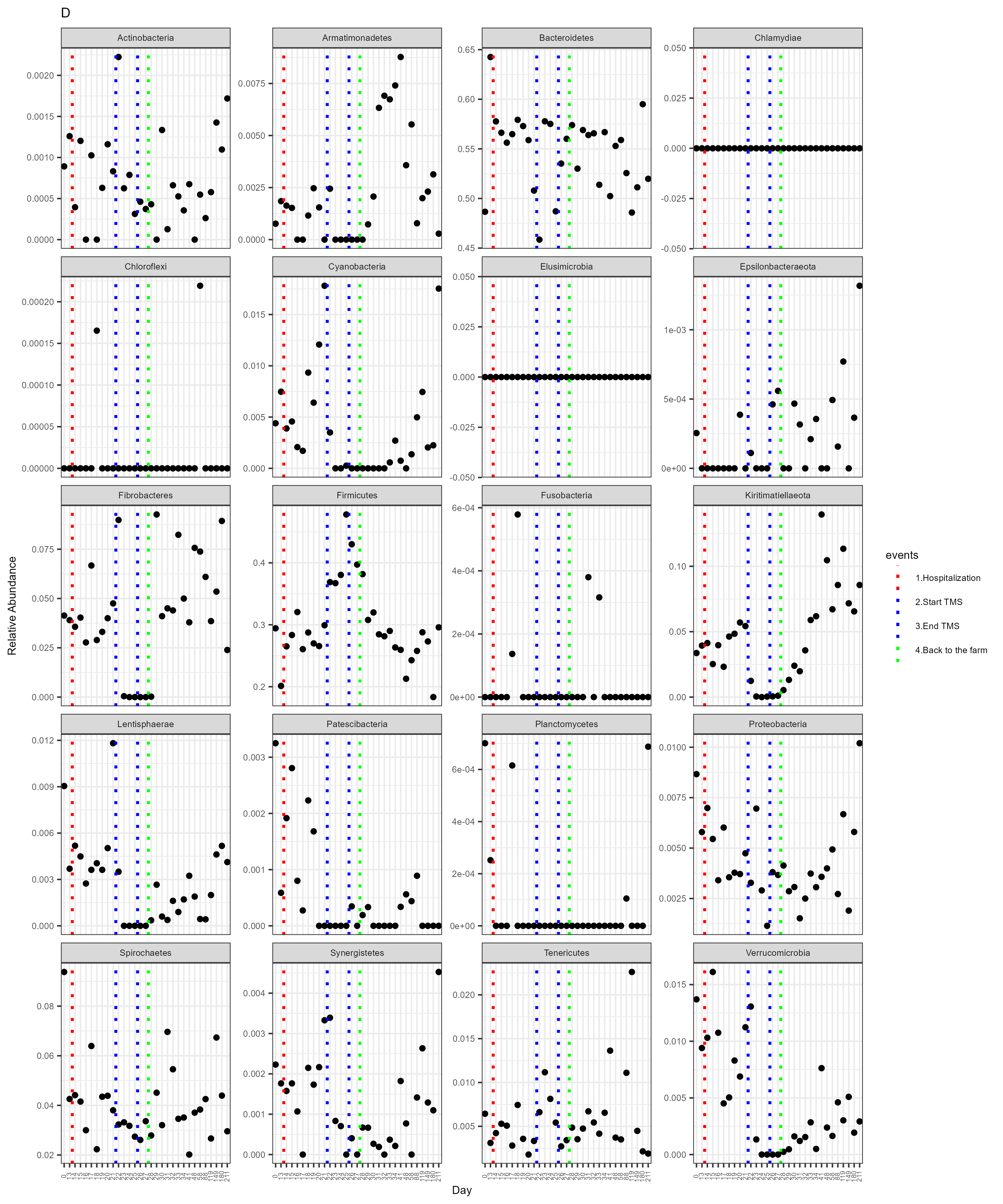
**

**E**

**
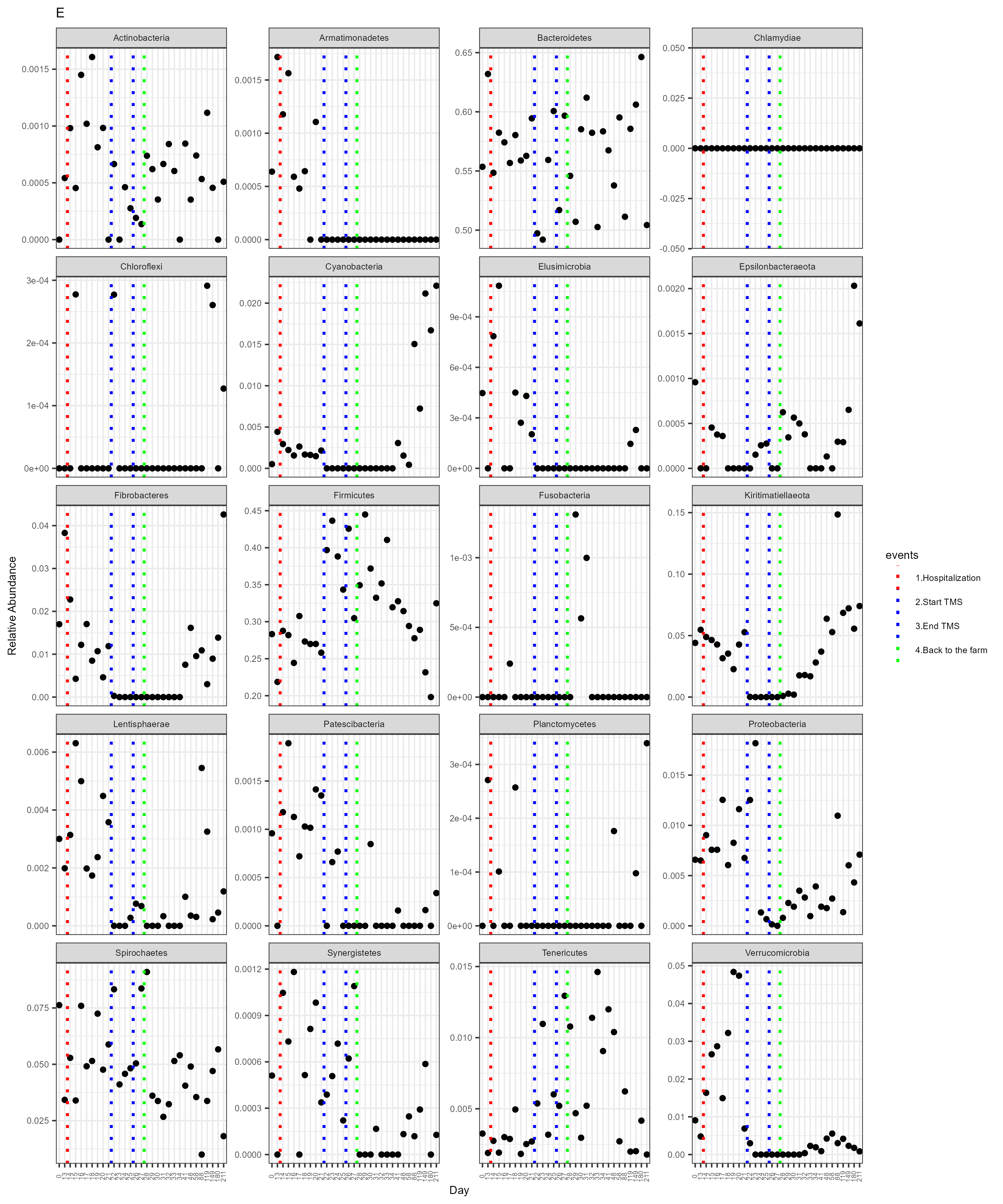
**

**F
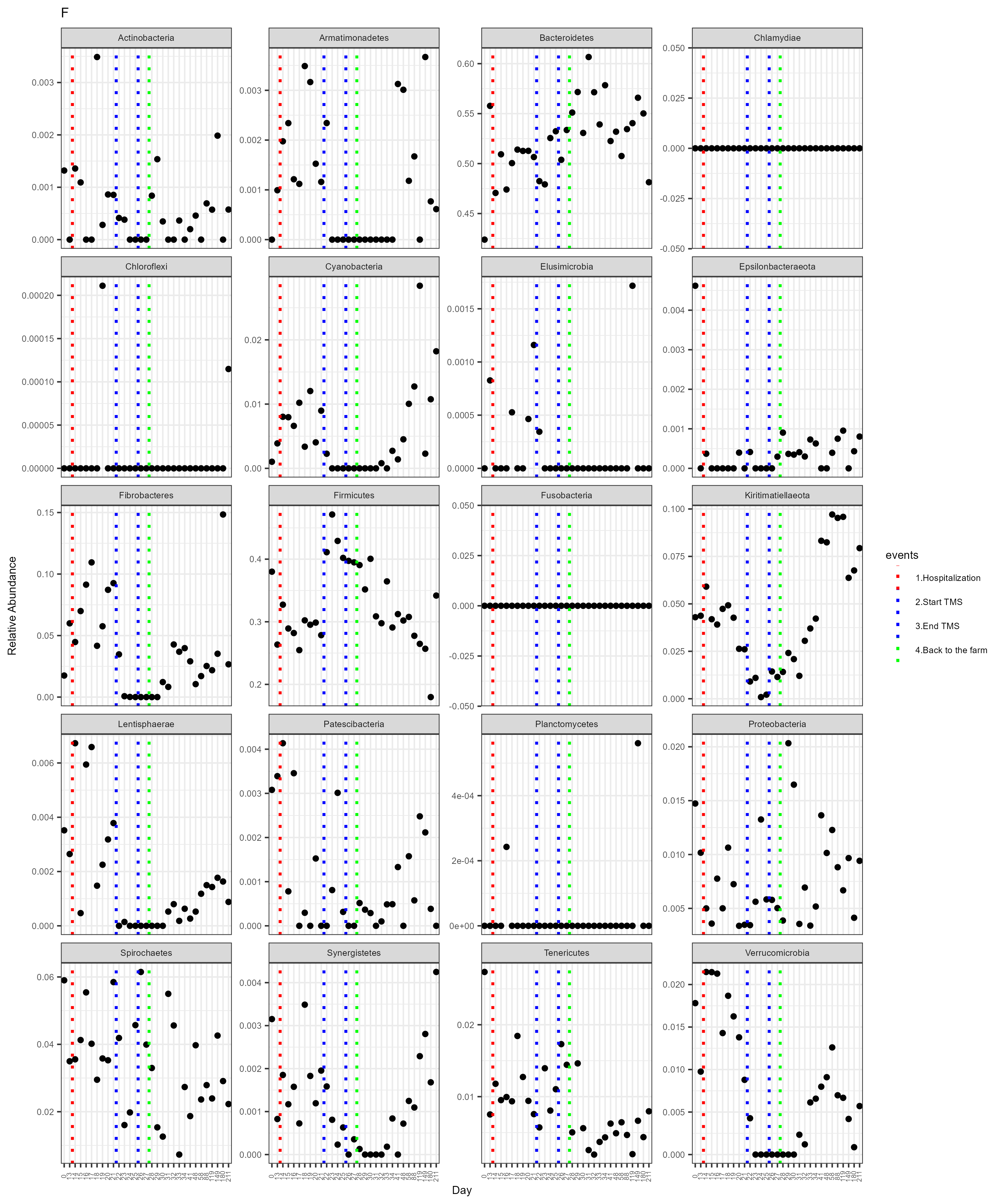
**

**Additional file 2.**

**Relative abundance of specified phyla at the level of the individual pony (A to F) over time*.*** *Relative abundance of phyla in the faecal microbiota of Welsh ponies at the farm (D0 - D13-1), during hospitalisation without treatment (D14 - D21), during hospitalisation and treatment with TMS (D22 - D26) and after discharge from the hospital up until six months after hospitalisation and antimicrobial treatment (D27 - D211). Scaling on the y-axis varies according to relative abundance.*
