# Supplementary material for: Longitudinal study of the short- and long-term effects of hospitalisation and oral trimethoprim-sulfadiazine administration on the equine faecal microbiome and resistome
Source: Microbiome. 2023 Feb 27;11:33. doi: 10.1186/s40168-023-01465-6 (PMC9969626; doi:10.1186/s40168-023-01465-6)
Supplement: Supplementary file 5 — Additional file 4. Relative abundance of bacterial families of the Bacteroidetes and Firmicutes phylum in faecal samples collected from Welsh ponies at the farm, during hospitalisation without treatment, during hospitalisation and treatment with TMS and after discharge from the hospital with six months follow-up. [file 40168_2023_1465_MOESM4_ESM.docx]

**Additional file 4.**

**Relative abundance of bacterial families of the Bacteroidetes and Firmicutes**

**A**

*
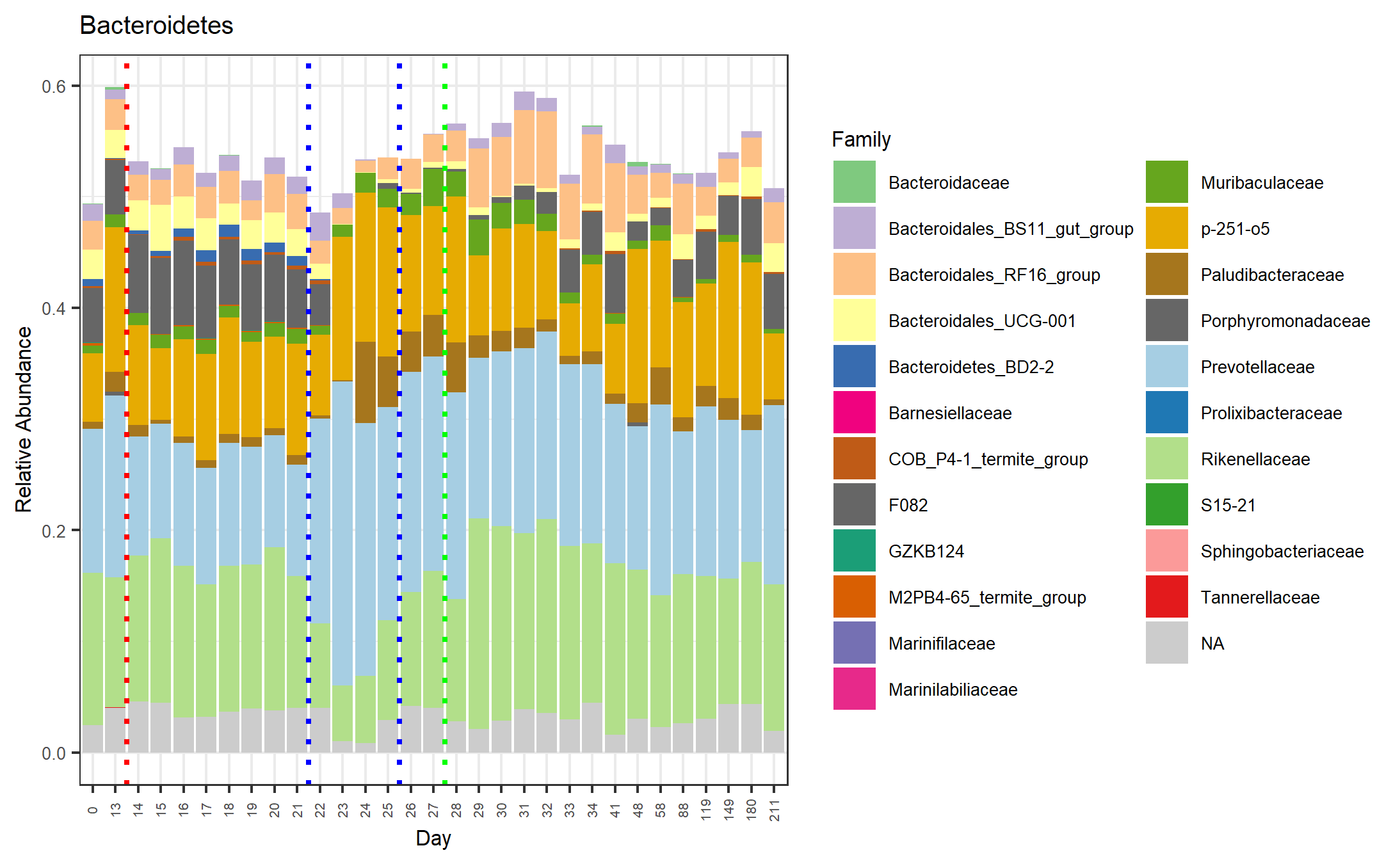
*

**B***
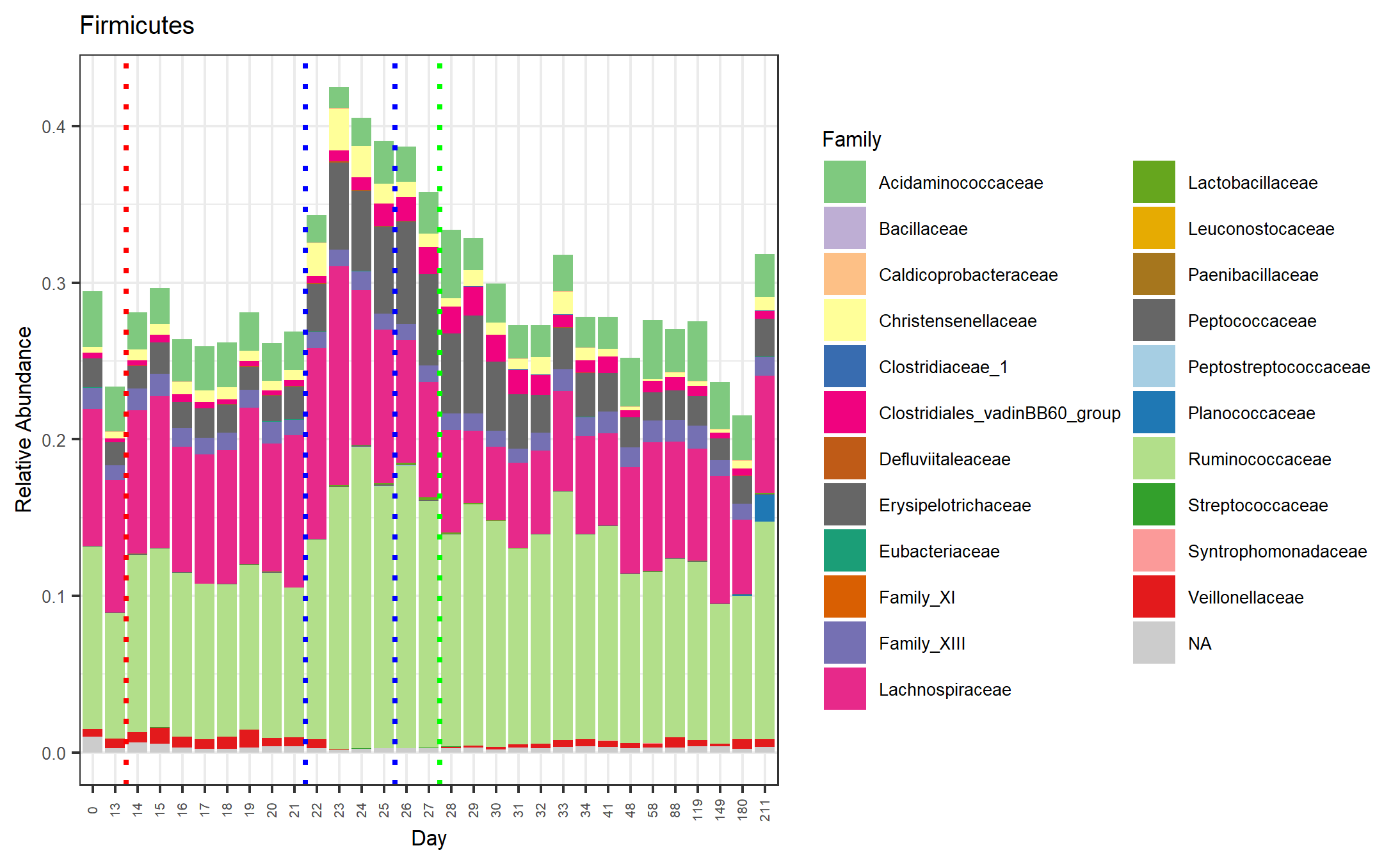
*

***Additional file 4. Relative abundance of bacterial families of the Bacteroidetes (A) and Firmicutes* *(B)*** *in faecal samples collected from healthy Welsh ponies at the farm (D0-D13), during hospitalization without treatment (D14-D21), during hospitalization and treatment with trimethoprim-sulfadiazine (TMS) (D22-D26) and after discharge from the hospital up until 6 months after hospitalization and antimicrobial treatment (D27-D211). NA = not applicable, indicating no family level name is yet available.*
