# Supplementary material for: Longitudinal study of the short- and long-term effects of hospitalisation and oral trimethoprim-sulfadiazine administration on the equine faecal microbiome and resistome
Source: Microbiome. 2023 Feb 27;11:33. doi: 10.1186/s40168-023-01465-6 (PMC9969626; doi:10.1186/s40168-023-01465-6)
Supplement: Supplementary file 6 — Additional file 5. Differentially abundant ASVs grouped by family at different time points during the study (before vs. after transportation; before vs. after hospitalisation for one week without antimicrobial treatment; before vs. after five days of treatment with TMS; the start vs. the end of the study). [file 40168_2023_1465_MOESM5_ESM.docx]

**Additional file 5.**

**Differentially abundant ASVs at different time points during the study.**

**A
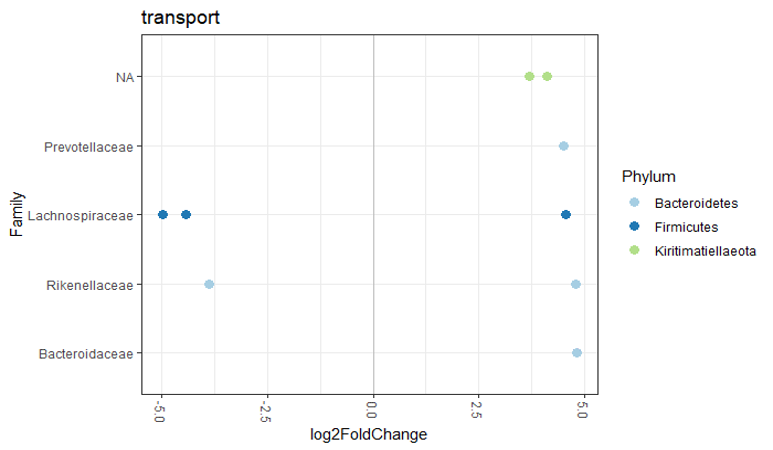
**

**B**

*
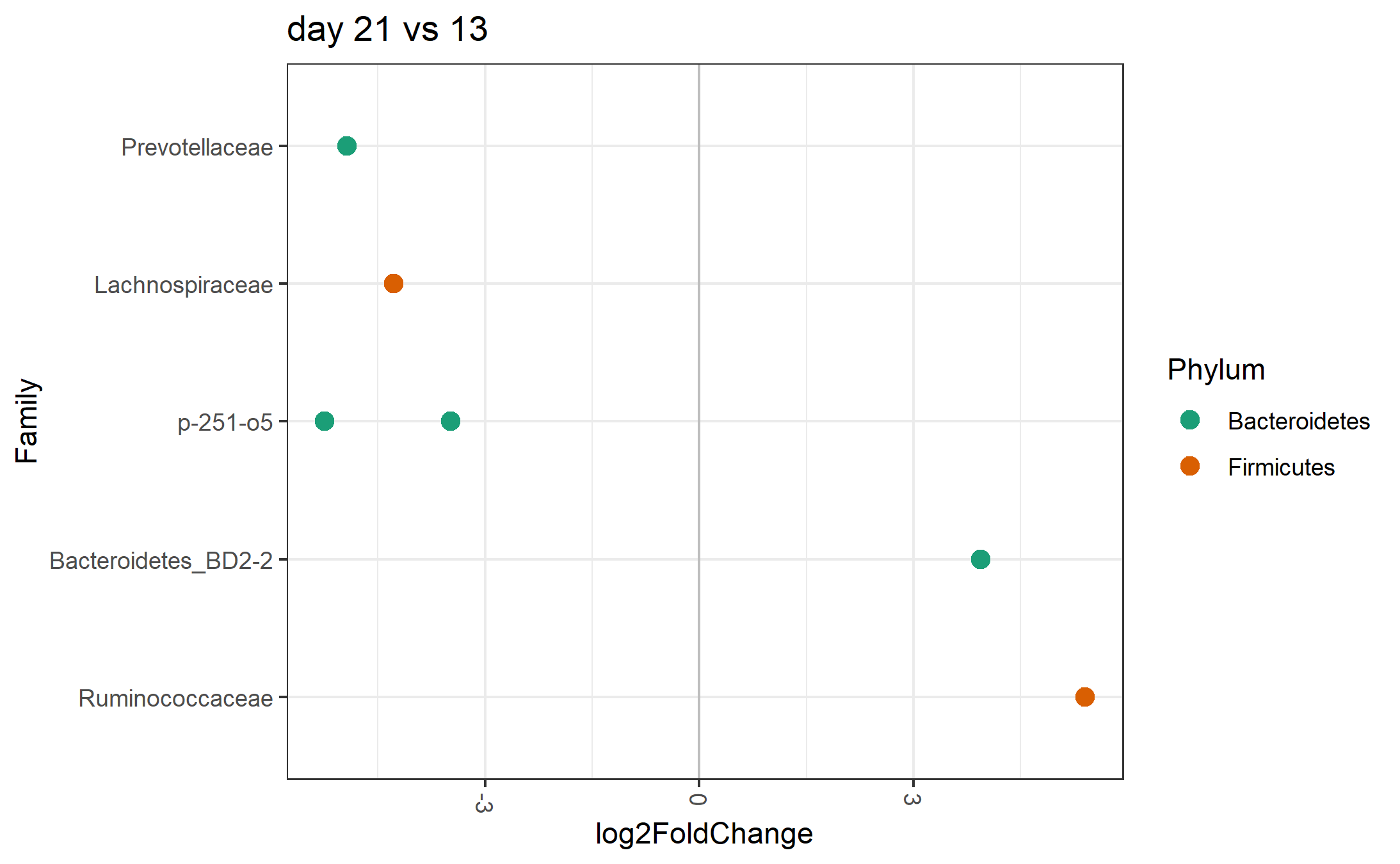
*

**C**

*
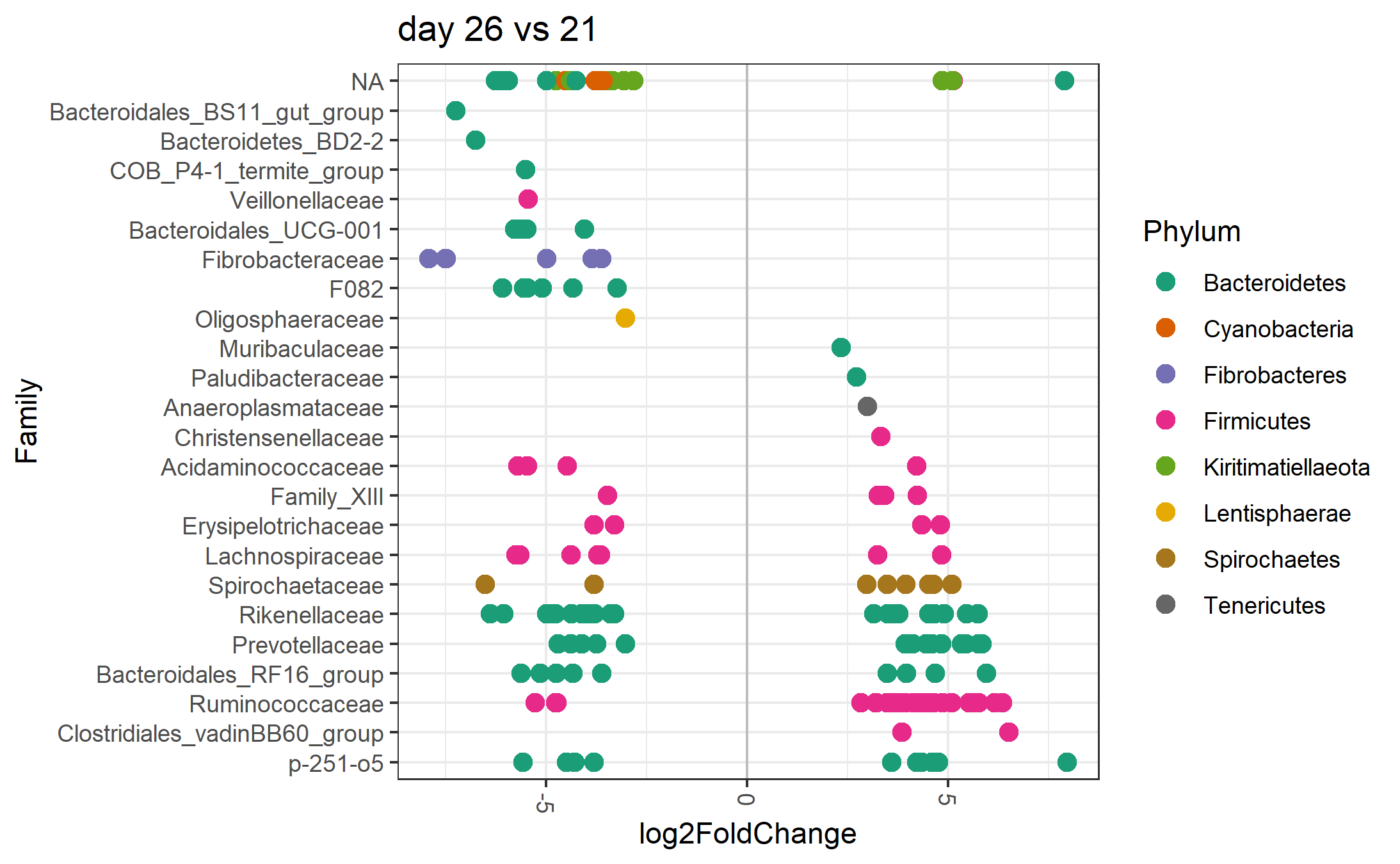
*

**D**

*
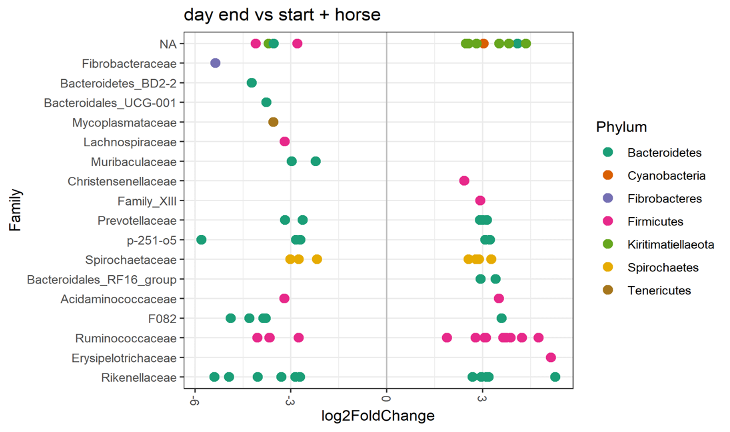
*

***Additional file 5.*** ***Differentially abundant ASVs at different time points during the study.*** Differentially abundant ASVs grouped by family: A) before (D13-1) and after transportation (D13-2) of 1,5h duration to the hospital B) before hospitalization (D13-1) and after one week of hospitalization without antimicrobial treatment (D21), C) before (D21) and after five days of treatment with TMS (D26) and D) at the start (D0-13-1) and the end (D180-211) of the study. For this last comparison (D) the variable ‘horse’ was included as covariate in the model since two samples were included in the start and end category instead of one (as was the case for all other comparisons). The log2 fold change in ASV abundance is shown on the x-axis. ASVs assigned to bacterial families on the left side of the plot are less abundant in samples collected at the later time point compared to earlier time point of sample collection. ASVs assigned to families depicted on the right side of the plot are more abundant in samples collected at the later time point compared to the earlier time point of sample collection. NA = ASV belonging to an unknown family (colours indicate the phylum).
